# TDAG51 is an ERK signaling target that opposes ERK-mediated HME16C mammary epithelial cell transformation

**DOI:** 10.1186/1471-2407-8-189

**Published:** 2008-07-02

**Authors:** Michael D Oberst, Stacey J Beberman, Liu Zhao, Juan Juan Yin, Yvona Ward, Kathleen Kelly

**Affiliations:** 1 Cell and Cancer Biology Branch, Center for Cancer Research, National Cancer Institute, National Institutes of Health, 37 Convent Drive, Room 1066, Bethesda, MD 20892, USA

## Abstract

**Introduction:**

Signaling downstream of Ras is mediated by three major pathways, Raf/ERK, phosphatidylinositol 3 kinase (PI3K), and Ral guanine nucleotide exchange factor (RalGEF). Ras signal transduction pathways play an important role in breast cancer progression, as evidenced by the frequent over-expression of the Ras-activating epidermal growth factor receptors EGFR and ErbB2. Here we investigated which signal transduction pathways downstream of Ras contribute to EGFR-dependent transformation of telomerase-immortalized mammary epithelial cells HME16C. Furthermore, we examined whether a highly transcriptionally regulated ERK pathway target, PHLDA1 (TDAG51), suggested to be a tumor suppressor in breast cancer and melanoma, might modulate the transformation process.

**Methods:**

Cellular transformation of human mammary epithelial cells by downstream Ras signal transduction pathways was examined using anchorage-independent growth assays in the presence and absence of EGFR inhibition. TDAG51 protein expression was down-regulated by interfering small hairpin RNA (shRNA), and the effects on cell proliferation and death were examined in Ras pathway-transformed breast epithelial cells.

**Results:**

Activation of both the ERK and PI3K signaling pathways was sufficient to induce cellular transformation, which was accompanied by up-regulation of EGFR ligands, suggesting autocrine EGFR stimulation during the transformation process. Only activation of the ERK pathway was sufficient to transform cells in the presence of EGFR inhibition and was sufficient for tumorigenesis in xenografts. Up-regulation of the PHLDA1 gene product, TDAG51, was found to correlate with persistent ERK activation and anchorage-independent growth in the absence or presence of EGFR inhibition. Knockdown of this putative breast cancer tumor-suppressor gene resulted in increased ERK pathway activation and enhanced matrix-detached cellular proliferation of Ras/Raf transformed cells.

**Conclusion:**

Our results suggest that multiple Ras signal transduction pathways contribute to mammary epithelial cell transformation, but that the ERK signaling pathway may be a crucial component downstream of EGFR activation during tumorigenesis. Furthermore, persistent activation of ERK signaling up-regulates TDAG51. This event serves as a negative regulator of both Erk activation as well as matrix-detached cellular proliferation and suggests that TDAG51 opposes ERK-mediated transformation in breast epithelial cells.

## Background

The development and progression of breast cancer is the result of multiple genetic changes, which lead to complex alterations in signal transduction networks in breast cancer cells relative to their normal epithelial counterparts. Signaling differences between tumor and normal cells are reflected in altered gene expression patterns, a finding that has been widely investigated using various molecular techniques. Patterns of differential gene expression have been used for classification and prognostication of certain cancers and may be valuable for prospectively predicting responsiveness to therapeutic treatments [1]. In addition to serving as biomarkers, some differentially expressed gene products have been informative in defining the physiological differences between normal and cancer cells. In order to understand the impact of dysregulated signaling pathways upon gene expression and function, it is necessary to connect differential gene expression to upstream signaling pathways.

Ras activation is a common intermediary in signaling pathways initiated by a variety of cell surface receptors, and signaling pathways downstream of Ras have been implicated repeatedly in oncogenesis. Ras proteins are frequently mutated to an activated form in human cancers, particularly in tumors of the pancreas, colon, thyroid, and lung [2]. Ras mutations are rare in breast cancer, where the mutation rate is less than 5% [3]. However, Ras signaling is hyperactive in many primary breast tumors [4]. This is most likely due to the activation of growth factor receptors that activate Ras exchange factors, such as the epidermal growth factor receptors EGFR (ErbB1) and ErbB2 (HER2/neu) and the colony stimulating factor-1 (CSF-1) receptor c-fms [5,6]. EGFR and ErbB2 receptor over-expression occurs in one quarter to one-half of all breast tumors, and this correlates with a significantly decreased disease-free and overall survival rates [5,7]. In addition, ligands for the EGFR such as transforming growth factor-a, amphiregulin, epiregulin, betacellulin, and heparin-binding EGF stimulate the receptor in primary breast tumors to enhance local growth and progression of the disease [8]. Finally, activating mutations in the PI3K pathway, an immediate downstream effector of Ras, occur in a subset of breast tumors [9,10]. Taken together, these findings suggest that breast cancer is a relevant model in which to study the biology of downstream Ras signal transduction.

The Ras oncogene binds numerous effectors that in turn activate a variety of signaling pathways. The most highly characterized of these are the Raf proteins, PI3K, and the Ral guanidine nucleotide exchange factors (RalGEFs), although Ras does recruit and activate other potentially transforming effectors [11]. The dissection of individual Ras signaling pathways is possible using effector domain mutants (EDMs) of Ras, for which single amino acid mutations in the effector binding domain allow the binding and activation of specific effectors, but not others [12]. These Ras EDMs, together with activated or dominant-negative versions of downstream effectors, have been used to characterize the pathways that contribute to the transformation of immortalized cells.

In mammary epithelial cells, both species- as well as cell line-dependent differences in Ras downstream signaling pathways have been found to induce tumorigenesis and/or anchorage-independent growth, a measure of *in vitro *transformation. Studies using immortalized *mouse *EpH4 mammary epithelial cells have implicated Raf as well as PI3K pathways in supporting transformation and tumorigenesis [13]. For *human *immortalized mammary epithelial cells, Raf and PI3K clearly contribute to transformation, although each is usually not sufficient for tumor formation in animal models [14,15]. In fact, the immortalized human breast epithelial cell line HMLE required simultaneous activation of Raf, PI3K, and the RalGEF pathways for maximal anchorage-independent growth and tumorigenic transformation [14].

Dissecting the physiological consequences of individual Ras-mediated signaling pathways with respect to mammary epithelial transformation is of obvious interest. The ability of activated Ras and Raf to induce autocrine expression of epidermal-like growth factors has been implicated in the protection of MCF10A mammary epithelial cells from anoikis [16]. Using HMEC16C cells, a telomerase-immortalized human mammary epithelial cell line, we have investigated the contribution of EGFR signaling to anchorage-independent growth initiated by Raf and additional signaling pathways downstream of Ras. We determined that ERK but not PI3K or RalGEF activation of HMEC16C cells supports anchorage independent proliferation independent of EGFR activation.

We performed a functional analysis of one gene in particular, TDAG51, whose expression is regulated by ERK through EGFR dependent and independent mechanisms. The loss of TDAG51 mRNA and protein has been correlated with breast adenocarcinoma and melanoma progression in clinical samples [17,18]. The importance of TDAG51 regulation on the transformed phenotype of Ras-infected cells was addressed using TDAG51-specific interfering small hairpin RNA (shRNA) to reduce TDAG51 levels. Consistent with a tumor suppressor role, loss of TDAG51 increased ERK-mediated cellular proliferation.

## Methods

### Culture of human epithelial cell lines

HME16C human mammary cells [19] were cultured in Clonetics Mammary Epithelial Basal Media (MEBM) with MEGM SingleQuot supplements (Cambrex), and HEK-HT human embryonic kidney epithelial cells [20] in DMEM (Invitrogen) plus 10% fetal bovine serum (Invitrogen). All cells were maintained at 37°C and 5% CO_2_. For induction of proteins from the tetracycline-inducible retroviral expression vector pLRT, 250 ng/mL of doxycycline (Sigma-Aldrich) was added to culture media.

### Retroviral and lentiviral constructs and infections

Constructs for the inducible expression of H-Ras, H-Ras effector domain mutants, and Rlf-CAAX were created by PCR subcloning the sequences of HA-tagged H-RasG12V (Ras^V12^), H-RasG12V, E37G (Ras^V12G37^), H-RasG12V, T35S (Ras^V12S35^), H-RasG12V, Y40C (Ras^V12C40^), and Rlf-CAAX [21] into the tetracycline-inducible retroviral expression vector pLRT [22]. The generation of retroviruses and lentiviruses was as described [23]. Polyclonal populations of pLRT infected cells were selected with 5 μg/mL blasticidin. The expression of the anti-TDAG51 shRNA 36-1 was accomplished by sub-cloning the anti-TDAG51 shRNA sequence 5'-GGAACTGCACTTCTCCAACTTCAAGAGAGTTGGAGAAGTGCAGTTCCTTTTT-3' into the pLVTHM lentivirus (kindly provided by Dr. D. Trono, Lausanne, Switzerland; [24]). After infection, cells with integrated lentivirus were selected by sterile sorting for GFP. Three sterile sorts were performed to acquire a polyclonal population of cells exclusively expressing GFP that was subsequently examined for TDAG51 protein reduction by western blotting.

### Western blotting and Ral activation assays

Western blots were performed using Chemilluminescence (Pierce). Antibodies used for western blots included anti-Ras mAb (BD Biosciences, cat#R02120), rat anti-HA mAb (Roche, cat#1988506), anti-phospho Erk mAb (Santa Cruz, cat#sc-7383), goat anti- Erk2 (Santa Cruz, C14-G, cat#154-G), rabbit anti-phospho-Akt Ser473 (Cell Signaling Technology), goat anti-Akt (Santa Cruz, C-20, cat#sc-1618), or an anti-alpha tubulin mAb (clone DM1A, NeoMarkers), followed by detection using appropriate anti-mouse, rabbit, or goat HRP-conjugated secondary antibodies (Jackson Labs) and ECL detection. For Ral activation assays, the Ral Activation Assay Kit (Upstate Cell Signaling Solutions) was used according to the manufacturer's protocol.

### Soft agar assays

Cells were trypsinized, neutralized, and 1.2 × 10^4 ^cells were plated per well in 0.36% bacto-agar (BD Biosciences), w/v in growth media, on a 0.6% bacto-agar support in 6-well culture plates. Prior to cell plating, 250 ng/mL doxycycline, DMSO vehicle, or the anti-EGFR compound PD153035 (Calbiochem), was added to the agar, as appropriate. Appropriately-supplememented fresh media was added to wells every 4 days. Colonies greater than 100 μm in diameter were counted after 18 days using a 2× lens equipped with a graded grid to determine colony size.

### Alamar Blue growth assays in ultra-low attachment tissue culture plates

Cells were trypsinized and plated in 500 μL appropriate growth media into 24-well ultra-low attachment plates (Corning) at 25,000 cells per well. At the times indicated, 50 μL of alamar blue (BioSource International) was added to each well and incubated for 6 hours under standard culture conditions. The amount of alamar blue reduction was quantified by measuring the fluorescence of each sample at 530 nm excitation/590 nm emission, corrected for the background fluorescence in wells containing media but no cells. The number of cells for initial plating and the alamar blue incubation times were determined by generating a standard curve of fluorescence versus cell number incubated with alamar blue for various times. Plating cells initially at 25,000/well insures that fluorescence measurements will occur on a linear part of the curve with a 6-hour incubation time over the course of an experiment. It was determined that TDAG51-specific shRNA did not affect the redox potential of HME16C at various cell numbers. Statistical analysis was performed using 2-way analysis of variance (ANOVA) with Bonferroni post-tests using the GraphPad Prism software program (GraphPad Software, Inc.).

### Tumorigenicity assays

Tumorigenicity assays were done essentially as described [23]. To induce gene expression, cells were treated with 250 ng/mL doxycycline three days prior to injection, and mice were fed with doxycycline-containing diet (200 mg/kg, BioServ, Inc.) beginning four days prior to cell inoculation and continuing for the duration of the experiment.

### Microarray analysis

Total RNA was extracted using Trizol reagent (Invitrogen) after treatment of cells with 250 ng/mL doxycycline for 72 hours to induce gene expression, or with doxycycline and 0.25 μM EGFR receptor kinase inhibitor PD153035 as indicated. Twenty micrograms of RNA were used for cDNA generation, and cDNA labeled with Cy3 or Cy5 monofunctional reactive dye to amino allyl-modified dUTP incorporated into cDNA using the FairPlay Microarray labeling kit (Stratagene). Labeled cDNA was hybridized to long-oligo (70-mer) cDNA microarrays from the NCI/CCR Microarray Center, NCI, Frederick, MD, according to standard protocols [25]. Hybridized arrays were analyzed using a GenePix 4000B array scanner and GenePix Pro 4.0 software (Axon Instruments, Molecular Devices Corporation). Data from GenePix Pro 4.0 was uploaded to the microarray database at the NCI/CCR Microarray Center website [25] for further analysis. Signal intensities of microarray features were calculated by subtracting the median local background from the median signal intensity. Features were considered for analysis if the signal intensity was greater than one standard deviation above background with at least a 2:1 signal-to-background ratio. Signal intensities for an entire microarray were normalized to the 50% percentile median value. After filtering and normalization, the Cy3 and Cy5 values were expressed as a ratio to indicate the fold up- or down-regulation. Two independent experiments for each comparison were performed, with a dye switch for each experiment, therefore yielding four separate data sets. For determining gene expression changes greater than or less than 2-fold, data sets were filtered for genes containing at least two significant values out of four array sets. Prior to filtering, all data points were analyzed using statistical analysis of microarray (SAM) data and a resultant gene set was chosen at a delta value of 0.4 that limited the false discovery rate (number of predicted false positive values/number of significant genes) for each analysis to less than 1%. Minimal information about a microarray experiment (MIAME)-compliant microarray data has been deposited with the National Center for Biotechnology Information (NCBI) Gene Expression Omnibus, accession number GSE8916, available at [26].

### Real-time RT-PCR analysis

cDNA was synthesized from RNA obtained for microarray analysis using the SuperScript™ III First-Strand Synthesis System for RT-PCR (Invitrogen). Quantification of relative cDNA levels for each gene was accomplished using the Platinum^® ^SYBR^® ^Green qPCR Supermix UDG real-time RT-PCR kit (Invitrogen) and a Rotor-Gene3000™ thermocycler with Rotor-Gene 5.0.37 software (Corbett Research) that calculates relative PCR synthesis rates by comparative quantification. The specificity of product synthesis was verified by melting curve analysis by the Rotor-Gene 5.0.37 software, and by running of real-time PCR products on 2% agarose gels to verify product size and rule out primer-dimer contribution to calculated values. The sequence-specific primers used in real-time RT-PCR can be found in Additional file 2.

### Cell cycle analysis

Cells grown under anchorage-independent conditions were spun down, washed once in sterile PBS, and suspended in a 50% mixture of PBS and ACCUMAX cell detachment solution (Chemicon International) for 10 minutes at 25°C to dissociate cell clumps. After dissociation, cells were washed once in cold PBS and then fixed with 70% ethanol. Fixed cells were treated with RNAse A for 20 minutes at 37°C, and nuclear DNA was stained with 50 μg/mL propidium iodide at 4°C. Cell cycle profiles were generated using a FACSCalibur flow cytometer (BD Biosciences) and modeling cell cycle phases with the cell cycle option of FlowJo flow cytometry software (Tree Star, Inc.) after gating for viable, single cells. Statistical analysis was performed using 2-way ANOVA with Bonferroni post tests using the GraphPad Prism software program (GraphPad Software, Inc.).

### EdU cell proliferation assay

Measurement of cell proliferation by 5-ethynyl-2'-deoxyuridine (EdU) incorporation was measured using the Click-iT EdU cell proliferation Assay Kit for Flow Cytometry (Invitrogen) according to the manufacturer's protocol. Briefly, cells were plated at 1 × 10^6 ^cells per well in 6-well ultra-low attachment plates or 5 × 10^5 ^cells per dish in 60 mm tissue culture dishes for attached control cells. Attached cells were treated with either DMSO or 10 μM EdU for 4 hours, and cells grown in low attachment plates were treated with DMSO or 10 μM EdU for 24 hours. Using FlowJo flow cytometry software (Tree Star, Inc.), DMSO-treated control cells were used to determine the threshold above which cells could be considered EdU positive, and the fraction of cells incorporating EdU was then determined for experimental samples.

### Cytotoxicity assay

The release of lactose dehydrogenase (LDH) into cell culture supernatant was measured using the cytotoxicity detection kit LDH (Roche Molecular Biochemicals) according to the manufacturer's instructions.

## Results

### Generation of H-Ras- and Rlf-CAAX-expressing HME16C cell lines

**Figure 1:**
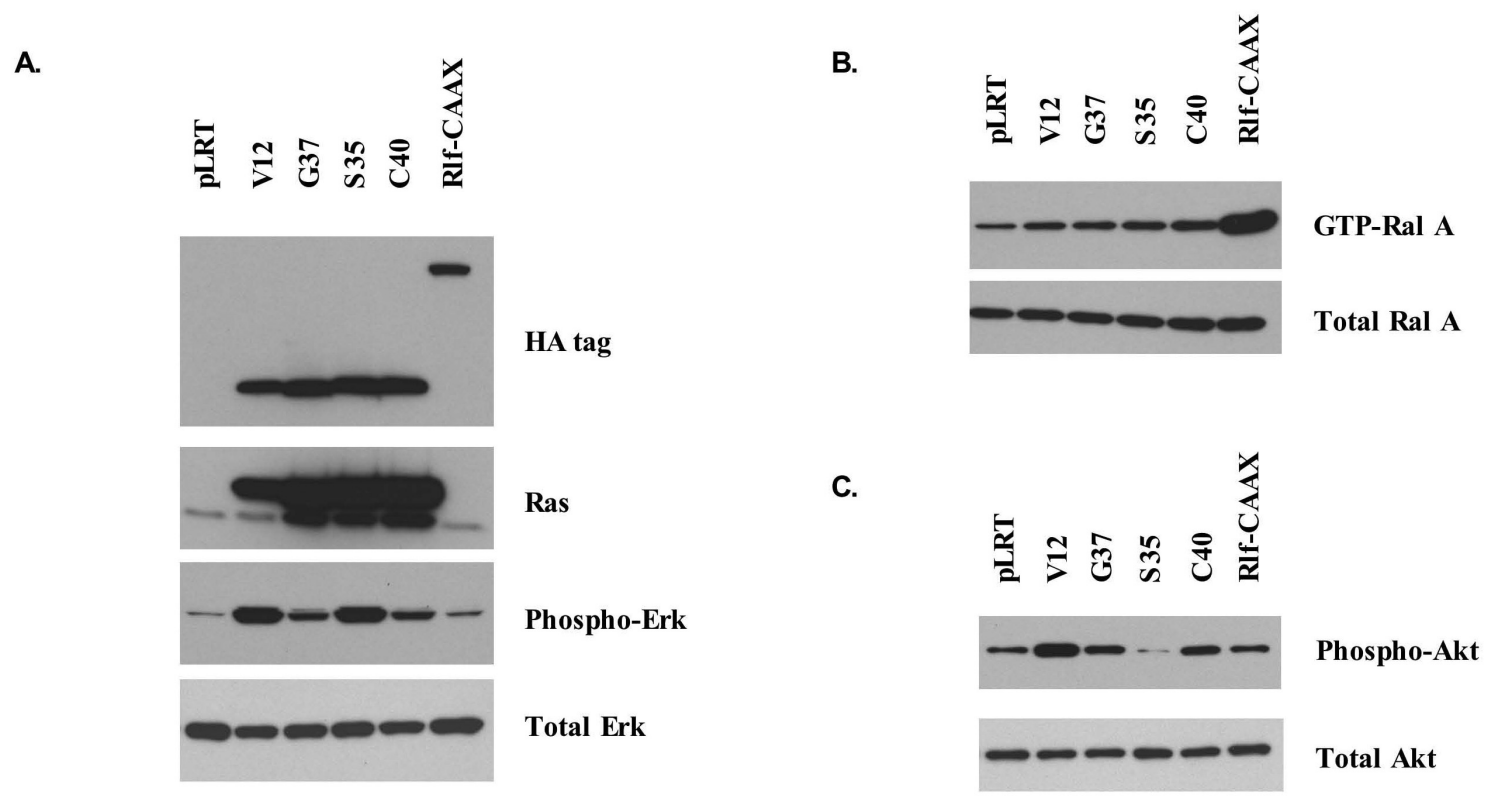
**Protein expression and pathway activation in HME16C cell lines**. (A) Expression of HA-tagged H-Ras^V12^, H-Ras^V12 ^effector domain mutants, and Rlf-CAAX in HME16C mammary epithelial cells. In this and subsequent figures, cells infected with the following vectors are indicated as follows: pLRT control (pLRT), H-Ras^V12 ^(V12), H-Ras^V12G37 ^(G37), H-Ras^V12S35 ^(S35), H-Ras^V12C40 ^(C40), and Rlf-CAAX (Rlf-CAAX). Cells were treated with 250 ng/mL doxycycline for 72 hours to induce gene expression, and anti-HA (HA tag) and anti-Ras (Ras) western blotting were performed. Erk activation was determined by western blotting for phosphorylated Erk (Phospho-Erk). The blot was subsequently stripped and probed with an anti-Erk2 antibody (Erk2). (B) Ral A activation was determined by a pull-down assay for GTP-bound, activated Ral A (GTP-Ral A). Total Ral A protein from lysates used in the Ral activation assay pull-down was determined by anti-Ral A western blotting (Ral A). (C) Akt activation in cell lysates was assessed by western blotting for phosphorylated Akt (Phospho-Akt). The membrane was stripped and probed with an anti-Akt antibody (Total Akt).

Retroviral vectors coding for amino terminal HA-tagged activated H-Ras^V12^, the H-Ras^V12 ^effector domain mutants (EDM) H-Ras^V12G37^, H-Ras^V12S35^, and H-Ras^V12C40^, and the constitutively activated version of a RalGEF, Rlf-CAAX, were used to infect telomerase immortalized HME16C human mammary epithelial cells. Anti-Ras and anti-HA western blotting demonstrated approximately equal levels of ectopic Ras expression among Ras-infected cells, with slightly lower levels in HME16C Ras^V12^-infected cells relative to EDM-infected cells (Figure 1A). Analysis of activated, GTP-bound Ral A demonstrated highly elevated levels of activated Ral A only in Rlf-CAAX-expressing cells and not in Ras-infected cells grown under standard culture conditions (Figure 1B). To assess activation of the ERK pathway, anti-phospho Erk western blotting was performed and showed significantly elevated Erk phosphoprotein in Ras^V12^- and Ras^V12S35^-infected HME16C cells relative to control pLRT-infected cells (Figure 1A). Elevated levels of phosphorylated Erk were also observed in Ras^V12G37^- and Ras^V12C40^-infected cells, although at a much lower level than that found in Ras^V12^- and Ras^V12S35^-infected cells. To assess activation of the PI3K signaling pathway, anti-phosphoAkt (Ser473) western blotting was performed to detect activated, phosphorylated Akt. In cells that were serum starved for 24 hours and matrix detached for 6 hours, elevated levels of phosphorylated Akt were observed in Ras^V12^-, Ras^V12C40^-, and Ras^V12G37^-infected HME16C, with highest levels present in Ras^V12^-infected cells (Figure 1C).

### Anchorage-independent growth of mammary epithelial cell lines

**Figure 2:**
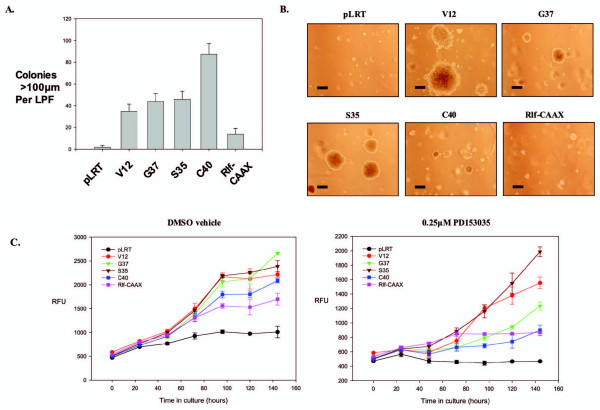
**Anchorage-independent growth of HME16C cell lines**. (A) Number of soft agar colonies exceeding 100 μM after 21 days of growth for HME16C cell lines infected with pLRT control, H-Ras^V12^, H-Ras^V12G37^, H-Ras^V12S35^, H-Ras^V12C40^, and Rlf-CAAX-expressing constructs. (B) Representative pictures of colony sizes in soft agar assays. Bar represents 200 μm. (C) Anchorage-independent growth of cell lines grown on ultra-low attachment plates treated with DMSO vehicle (left) or 0.25 μM PD153035 (right) measured using the alamar blue assay. RFU, relative fluorescence units of alamar blue product formed.

To assess transformation by different Ras signaling pathways, anchorage-independent growth assays were performed in soft agar and in ultra-low attachment tissue culture plates. Ras- and Ras EDM-infected HME16C cells formed significantly more soft agar colonies >100 μm in diameter than pLRT vector-infected cells (Figure 2A). The Ras^V12^-infected cells formed large colonies, many exceeding 1000 μm, although the total number exceeding 100 μm was typically less than that for the Ras EDM (Figure 2B). Among the Ras EDM-infected cells, the Ras^V12S35^-infected cells formed the largest colonies. These were similar to, but smaller than, the Ras^V12^-infected cells. For colonies above 100 μm in diameter, Ras^V12C40^-infected cells were the most efficient at colony formation, despite the smaller mean size of colonies. Rlf-CAAX-infected cells formed slightly more colonies above 100 μm than vector-transfected control cells, but these were significantly smaller than those formed by Ras- and Ras EDM-infected cells.

When grown under anchorage-independent conditions in ultra-low attachment plates, the accumulation of cells for the various infected cell lines roughly paralleled the total cell masses seen in soft agar growth assays (Figure 2C). The Ras^V12^- and Ras^V12 ^EDM-expressing cells grew well, while the growth of the Rlf-CAAX-expressing cells was significantly less. The HME16C cells maintained viability but did not increase in number.

To compare our results to others, we verified the function of pLRT vector driven Ras EDM mutants and Rlf-CAAX in HEK-HT cells, which previously have been reported to form colonies in soft agar upon expression of H-Ras^V12G37 ^and Rlf-CAAX [27]. In our hands, expression of H-Ras^V12^, H-Ras^V12G37^, and Rlf-CAAX from the pLRT vector induced efficient soft agar colony growth, and H-Ras^V12S35 ^and H-Ras^V12C40 ^did not (not shown), identical to previously reported results [27]. Expression of exogenous H-Ras and Rlf-CAAX, and activation of endogenous RalA, in this cell line was similar to that observed in HME16C cells (not shown). Therefore, expression of Rlf-CAAX and subsequent RalA activation did not appear to be sufficient to induce anchorage-independent growth in the HME16C mammary epithelial cell line in contrast to HEK-HT and various other immortalized human cell types [27].

### Tumorigenesis of HME16C cell lines in nude mice

Anchorage-independent growth often predicts the ability of cells to grow as xenografted tumors in immuno-compromised mice. Tumor formation was assessed following subcutaneous inoculation. The Ras^V12^-infected HME16C cells formed rapidly growing, fluid-filled tumors with an average latency of 4 weeks and a mean tumor volume of 808.8 mm^3 ^at 6 weeks (Table 1). Approximately one-half of the tumors were aspirated prior to sacrifice, and a sero-sanguinous fluid was observed, on average accounting for roughly one-third of the measured tumor volume. Histological analysis of H&E stained tumor sections revealed poorly differentiated spindle-shaped tumor cells with prominent squamous cell differentiation and extracellular keratin deposition (not shown). Tumors also contained a strong inflammatory component. Of the other cell lines tested, only the Ras^V12S35^-infected HME16C cells formed palpable tumors in 50% of injected animals with an average latency of approximately 12 weeks and a mean tumor volume of 109.0 mm^3 ^at 16 weeks, considerably smaller than Ras^V12^-expressing tumors (Table 1). Cells within Ras^V12S35^-infected tumors resembled the histology of Ras^V12 ^tumor cells but with less keratin deposition and without the formation of fluid-filled spaces (not shown). Empty vector-, Ras^V12G37^-, Ras^V12C40^-, and Rlf-CAAX-infected cells failed to form palpable tumors four months after injection. The metastatic potential of Ras^V12^- and Ras^V12G37^-expressing cell lines was tested by tail-vein injection in nude mice, but no metastatic lesions were observed by histological analysis in lungs, liver, spleen, or kidneys at 16 weeks post injection (not shown).

**Table 1:** Growth of HME16C tumors in nude mice

Cell Line Innoculated	Number of Mice with Tumors	Mean Tumor Latency	Mean Tumor Volume at Sacrifice (mm^3^)*
pLRT	0/10	-	-
H-Ras^V12^	10/10	4 weeks	808.8 +/- 635.2
H-Ras^V12G37^	0/10	-	-
H-Ras^V12S35^	5/10	12 weeks	109.0 +/- 171.0
H-Ras^V12C40^	0/10	-	-
Rlf-CAAX	0/10	-	-

Cells infected with control, Ras-expressing, or Rlf-CAAX-expressing retrovirus were injected subcutaneously into nude mice and examined for tumor formation and growth. *For mice injected with H-Ras^V12S35 ^cells, includes only sites that formed tumors.

### Autocrine EGFR signaling is required for Ras^V12G37^- and Ras^V12C40^-mediated, but not Ras^V12S35^-mediated, HME16C cell anchorage- independent growth

**Figure 3:**
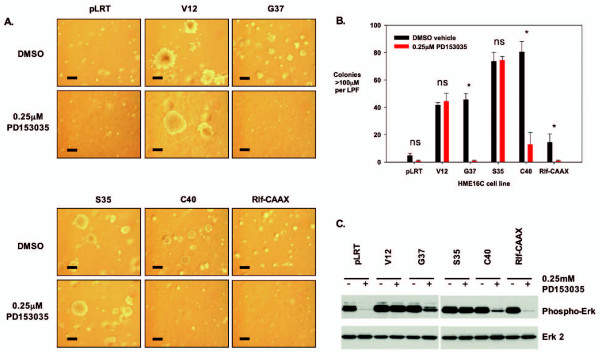
**Anchorage-independent growth of HME16C cell lines after EGFR inhibition**. (A) Cell lines, as indicated, were grown in the soft agar assay for 21 days either in the presence of DMSO vehicle or 0.25 μM PD153035 EGFR inhibitor. Scale bar, 200 μm. (B) Number of colonies >100 μm in size for each cell line treated with either DMSO vehicle or 0.25 μM of the EGFR-specific inhibitor PD153035. Statistical analysis was performed using one-way analysis of variance with Bonferroni post-test analysis. NS, not significant, p > 0.05; *, p < 0.0001. (C) Activation of Erk after EGFR inhibition. Cell lines were grown in the presence of DMSO vehicle or 0.25 μM PD153035 for 48 hours, followed by western blotting for phosphorylated Erk (Phospho-Erk). The blot was stripped and probed with an anti-Erk 2 antibody (Erk 2) to verify equal protein loading. The phospho-ERK blot was overexposed in order to reveal minimal levels of protein.

EGFR signaling is frequently altered in breast cancer, where EGFR and ErbB2 over-expression are common events. cDNA microarray and real-time RT-PCR analysis of HME16C Ras^V12^- and Ras EDM-infected cells (Additional file 1) revealed increased levels of mRNAs for EGFR ligands, including epiregulin, amphiregulin, and TGFa (Table 2). In addition, increased levels of phospho-Erk were unexpectedly seen in Ras^V12G37^- and Ras^V12C40^-infected cells, possibly the result of autocrine activation of endogenous EGFR by secreted EGFR ligands. The presence of EGFR was established using Western blots (not shown). Therefore, we sought to determine if autocrine signaling by the EGFR was required for the anchorage-independent growth of Ras- or Ras EDM-infected cells. Ras^V12^- and Ras EDM-infected HME16C cells were grown in soft agar in the presence or absence of the EGFR-specific inhibitors, PD153035 and PD168393. Complete inhibition of colony formation for Ras^V12G37^-infected and Ras^V12C40^-infected cells was observed at 0.25 μM PD153035, a concentration that specifically inhibits EGFR [28], whereas both the Ras^V12^- and Ras^V12S35^-infected cells formed colonies efficiently at this same concentration of inhibitor (Figure 3A and 3B). Identical results were found for the EGFR-specific inhibitor PD168393 used at 0.1 μM, a concentration that specifically inhibits EGFR and Her-2 receptors [29](not shown). Similarly, treatment of cells grown in ultra-low-attachment plates also demonstrated that EGFR inhibition substantially inhibited growth of Ras^V12G37^-and Ras^V12C40^-expressing cells relative to that of Ras^V12^- and Ras^V12S35^-expressing HME16C (Figure 2C). Western blotting of cellular lysates from cells treated with 0.25 μM PD153035 showed that high levels of phosphorylated Erk were maintained only in Ras^V12^- and Ras^V12S35^-infected cells, but were significantly reduced in Ras^V12G37^- or Ras^V12C40^-infected cells treated with the inhibitor (Figure 3C), while phoshorylated Akt was minimally affected (not shown). Anchorage-independent growth therefore correlated with maintenance of high Erk activity in HME16C cells. Consistent with this observation, inhibition of MEK, and therefore ERK signaling, using the MEK-specific inhibitor PD98059 at 10 μM, significantly inhibited soft agar colony formation by all cell lines (not shown).

**Table 2:** Up-regulation of EGFR ligands in Ras^V12^- and Ras EDM-expressing HME16C

Gene	V12	S35	G37	C40
epiregulin	**2.6 **(7.0)	**1.9 **(1.0)	**1.8 **(2.4)	**1.2 **(1.3)
TGFa	**3.5 **(3.4)	**2.9 **(1.3)	**-1.1 **(-1.4)	**1.9 **(-1.1)
amphiregulin	**1.4 **(2.4)	**2.9 **(1.0)	**2.3 **(1.7)	**2.2 **(1.0)

Fold up-regulation of the EGFR ligands epiregulin, transforming growth factor alpha (TGFa), and epiregulin in Ras^V12^- and Ras EDM-expressing HME16C as determined by real-time RT-PCR quantification (boldface) and cDNA microarray analysis (in parentheses).

### Microarray analysis of gene expression changes in Ras^V12^-, Ras^V12G37^-, Ras^V12S35^-, and Ras^V12C40^-infected HME16C cells

Activation of the Ras oncogene is accompanied by the stimulation of multiple signal transduction pathways leading to the activation or repression of numerous transcription factors as well as changes in mRNA translation and stability, and thus, the modulation of gene expression. To determine which gene expression changes accompany the transformation of HME16C human epithelial cells by activated Ras, we examined our transformed HME16C cells by cDNA microarray analysis. To do this, RNA was isolated from H-Ras^V12 ^and H-Ras^V12 ^EDM expressing cells after treatment with doxycycline to fully induce gene expression and compared to RNA from identically treated pLRT vector-infected control cells. Statistical analysis of microarray (SAM) data analysis was performed for the datasets, and a delta value of 0.4 was chosen for each dataset, which maintains the estimated false discovery rate (FDR) below 1% for each. A summary of the genes up- or down-regulated greater than 2-fold in the H-Ras^V12^- and Ras effector domain mutant-infected HME16C cell lines is presented in Additional file 1, organized according to broad categories of gene function. To validate gene expression changes identified by cDNA microarray analysis, quantitative RT-PCR was performed using RNA from the same samples used in microarray analysis, and is presented in Additional file 2. Results for 22 of 26 genes chosen to reflect genes up- or down-regulated both strongly or weakly showed strong agreement with microarray data, demonstrating that the microarray dataset represents a reliable quantification of gene expression changes.

To evaluate the effect of EGFR inhibition on gene expression, Ras^V12^-, Ras^V12S35^-, and Ras^V12G37^-infected cells were induced with doxycycline and subsequently incubated either in the presence or absence of 0.25 μM PD153035, and microarray analysis comparisons were made to vehicle-treated pLRT-infected cells. Almost all Ras and Ras EDM-induced upregulated transcriptional responses were blocked by pharmacological inhibition of EGFR, consistent with previous reports for inhibition of Raf-regulated transcription [30]. Our analysis identified PHLDA1 as an up-regulated gene in both vehicle-treated and PD153035-treated Ras^V12 ^and Ras^V12S35 ^cells, although the relative fold increase was reduced following EGFR inhibition. By comparison, PHLDA1 was down-regulated in PD153035-treated Ras^V12G37 ^relative to vehicle-treated cells (not shown). Therefore, PHLDA1 represents a Raf/ERK-responsive gene whose expression parallels EGFR-independent HME16C mammary epithelial cell transformation.

### TDAG51 expression is up-regulated by Ras signaling in a ERK-dependent manner, and is associated with EGFR-independent transformation

**Figure 4:**
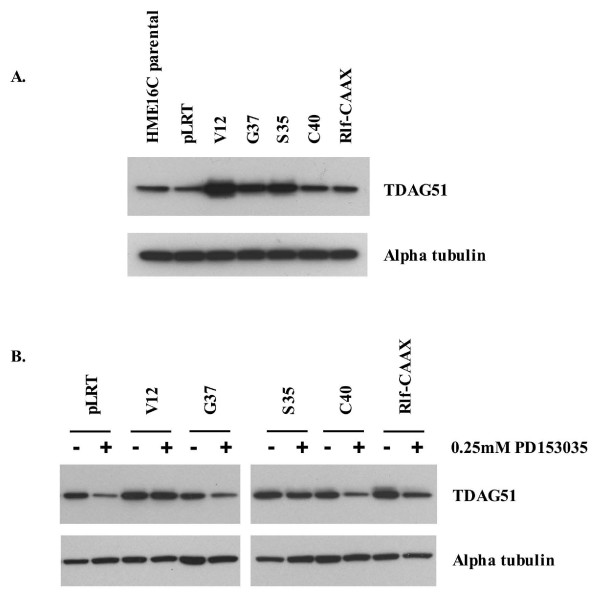
**Expression of TDAG51 in HME16C cell lines in the absence or presence of the EGFR inibition**. Western blotting of TDAG51 in HME16C cell lines. (A) Twenty-five micrograms of protein from HME16C cellular lysates were resolved by SDS-PAGE and probed with an anti-TDAG51 monoclonal antibody (TDAG51). Equal protein loading was verified by stripping the blot and probing with an alpha tubulin monoclonal antibody (Alpha tubulin). Band intensities were determined using Image J software (NIH, Bethesda, MD) and TDAG51 values were normalized using alpha tubulin values. Fold expression of each band relative to HME16C parental: HME16C parental, 1.0; pLRT, 1.0; V12, 3.0; G37, 2.3; S35, 2.4; C40, 1.3; Rlf-CAAX, 0.9. Similar experiments to that shown were performed 2 additional times with similar results. (B) HME16C cell lines were treated with DMSO vehicle or 0.25 μM PD153035 EGFR-specific inhibitor for 48 hours, and cell lysates were prepared. Twenty five micrograms of protein were resolved by SDS-PAGE and western blotted with anti-TDAG51 mAb (TDAG51). The blot was subsequently stripped and probed with an alpha tubulin mAb (Alpha tubulin). Band intensities were determined using Image J software (NIH, Bethesda, MD) and TDAG51 values were normalized using alpha tubulin values. DMSO-treated cells are indicated by a -, and PD1535035-treated cells are indicated by a +. Fold expression relative to DMSO-treated pLRT: pLRT -(1.0), +(0.2); V12 -(2.3), +(2.6); G37 -(0.8), +(0.3); S35 -(1.4), +(0.9); C40 -(1.1), +(0.6); Rlf-CAAX -(1.6), +(1.0). Similar experiments to that shown were performed 2 additional times with similar results.

**Figure 5:**
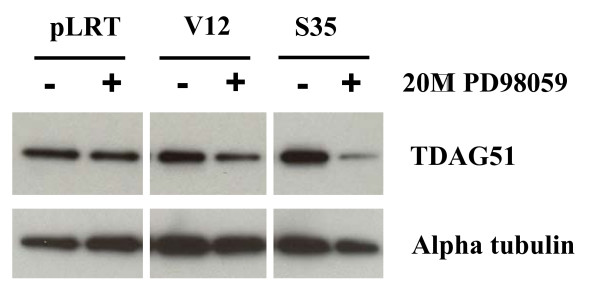
**TDAG51 expression is induced by ERK pathway activation**. HME16C cell lines infected with pLRT empty vector (pLRT), Ras^V12 ^(V12), or Ras^V12S35 ^(S35) were treated with 0.2% DMSO vehicle or 20 μM PD98059 for 24 hours, followed by western blotting for TDAG51. The blot was stripped and probed with an alpha tubulin monoclonal antibody (Alpha tubulin) to demonstrate equal protein loading. Band intensities were determined using Image J software (NIH, Bethesda, MD) and TDAG51 values were normalized using alpha tubulin values. Fold expression of PD98059-treated relative to DMSO-treated contols: pLRT (0.6); V12 (0.6); S35 (0.3). Similar experiments to that shown was performed 1 additional time with similar results.

The PHLDA1 gene is of interest as it has been suggested to be a tumor suppressor in breast adenocarcinoma and melanoma [17,18]. We further analyzed the signal dependent expression of the PHLDA1 gene and its protein product, TDAG51. Microarray analysis identified the PHLDA1 gene as being dramatically up-regulated in Ras^V12^- and Ras EDM-infected cells to levels that correlated with the level of ERK activation and the extent of anchorage-independent growth (Additional file 1). Western blotting confirmed that TDAG51 was also upregulated in a similar manner (Figure 4A). The PHLDA1 gene was elevated in PD153035-treated Ras^V12^- and Ras^V12S35^-infected cells but was significantly dependent upon EGFR tyrosine kinase activity for upregulation in Ras^V12G37^- and Ras^V12C40^-infected cells (not shown), and the expression of the encoded TDAG51 protein approximately paralleled PHLDA1 RNA expression (Figure 4B). As shown in Figure 3C, EGFR inhibition significantly reduced ERK signaling in Ras^V12G37^- and Ras^V12C40^-infected cells without affecting Ras^V12^- and Ras^V12S35^-infected cells. To confirm that TDAG51 up-regulation was induced specifically by ERK activation, we treated pLRT-, Ras^V12^-, and Ras^V12S35^-infected cells with the MEK-specific inhibitor PD98059. PD98059 used at 20 μM appears to be specific for MEK1 as it does not nonspecifically inhibit a variety of other protein kinases that have been assayed. [31]. TDAG51 up-regulation was attenuated by MEK inhibition (Figure 5). TDAG51 therefore represents an ERK-inducible gene whose up-regulation in HME16C is correlated with an EGFR-independent, ERK-mediated transformation.

### TDAG51 up-regulation opposes ERK-mediated HME16C transformation

**Figure 6:**
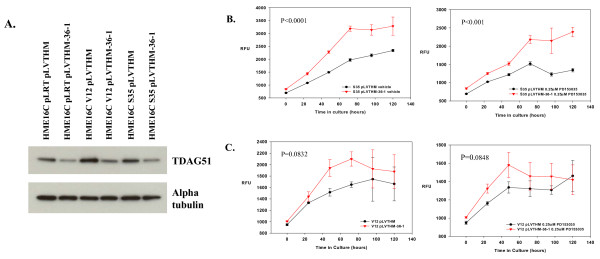
**Reduction of TDAG51 expression in Ras^V12 ^and Ras^V12S35^-infected cells and their anchorage-independent growth**. (A) Western blotting of TDAG51 protein (TDAG51) from 5 μg of cellular lysates from non-silencing vector control (pLVTHM) and anti-TDAG51 shRNA (36-1) -expressing cell lines as indicated. The blot was subsequently stripped and probed with an anti-alpha tubulin mAb (Alpha tubulin). Band intensities were determined using Image J software (NIH, Bethesda, MD) and TDAG51 values were normalized using alpha tubulin values. Fold expression of each band relative to HME16C pLRT pLVTHM: pLRT pLVTHM (1.0), 36-1 (0.4); V12 pLVTHM (2.3), 36-1 (1.0); S35 pLVTHM (1.5), 36-1 (0.7). Similar experiments to that shown were performed 2 additional times with similar results (B) Growth of pLVTHM vector control or TDAG51 shRNA-expressing Ras^V12S35^-transduced cells either in the presence of 0.2% DMSO control (left) or 0.25 μM PD153035 (right) in low-attachment plates, assessed by the alamar blue growth assay. RFU, relative fluorescence units. (C) Growth of pLVTHM vector control or TDAG51 shRNA-expressing Ras^V12^-transduced cells either in the presence of 0.2% DMSO control (left) or 0.25 μM PD153035 (right) in ultra-low-attachment plates, assessed by the alamar blue growth assay. RFU, relative fluorescence units.

To analyze the role of TDAG51 in ERK-dependent growth, we reduced TDAG51 expression in Ras^V12^- and Ras^V12S35^-infected cells to a level comparable to that in non-transformed vector-infected control cells using stably expressed TDAG51-specific shRNA (Figure 6A). Cell proliferation of attached cells grown on tissue culture plastic was unaffected by lowered TDAG51 protein levels (not shown). However, cell growth under anchorage-independent conditions in ultra-low attachment plates was significantly enhanced by TDAG51 knock-down in Ras^V12S35^-infected cells (Figure 6B). Likewise, results with Ras^V12^-infected cells stably infected with TDAG51-targeting shRNA also showed enhanced growth relative to vector-infected control cells, although to a lesser extent than that seen with Ras^V12S35^-infected cells (Figure 6C). This suggests that Ras signaling pathways other than ERK compensate partially for the negative growth effects of TDAG51, or that Ras^V12^-infected cells are already close to maximally transformed under anchorage-independent growth conditions.

### Loss of TDAG51 expression in transformed cells stimulates both cell cycle progression and apoptosis

**Figure 7:**
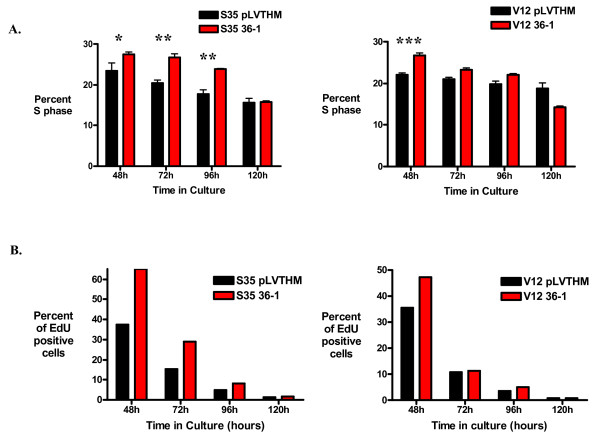
**Cell proliferation is increased in Ras-transformed cell lines with reduced TDAG51 expression**. (A) S phase fraction of Ras^V12S35^-expressing (left panel) or Ras^V12^-expressing (right panel) HME16C infected with pLVTHM non-silencing vector control or anti-TDAG51 shRNA 36-1 after 48, 72, 96 or 120 hours growth in ultra-low attachment plates as determined by cell cycle analysis of propidium iodide-stained nuclei. * p < 0.05; **p < 0.01; ***p < 0.001. (B) Incorporation of the nucleoside analogue EdU into cellular DNA measured in Ras^V12S35^-expressing (left panel) or Ras^V12^-expressing (right panel) HME16C infected with pLVTHM non-silencing vector control or anti-TDAG51 shRNA 36-1 after 48, 72, 96 or 120 hours growth in ultra-low attachment plates. Incorporated EdU was quantified by detection of Alexa Fluor 488-labeled EdU in viable single cells by flow cytometry analysis.

To characterize the effect of TDAG51 on cell proliferation under anchorage-independent conditions, cell cycle analysis and cell proliferation assays for Ras^V12S35^- and Ras^V12^-infected cells were performed. Both Ras^V12S35^- and Ras^V12^- TDAG51 shRNA-expressing cells demonstrated an increased S-phase fraction versus pLVTHM vector control cells at various time points during anchorage-independent growth (Figure 7A). In concordance with these results, Ras^V12S35 ^and Ras^V12 ^cells expressing the TDAG51-specific shRNA showed enhanced incorporation of 5-ethynyl-2'-deoxyuridine (EdU) in cell proliferation assays, indicating a higher rate of DNA synthesis in cells with reduced TDAG51 protein (Figure 7C).

**Figure 8:**
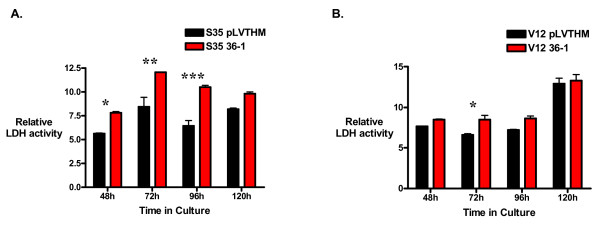
**LDH release by Ras^V12S35^- or Ras^V12^-expressing HME16C infected with pLVTHM vector or anti-TDAG51 shRNA 36-1**. (A) Relative LDH activity released by pLVTHM- or anti-TDAG51 shRNA 36-1-infected Ras^V12S35 ^cells was measured in the culture media of cells grown under anchorage-independent conditions in ultra-low attachment plates for the times indicated. * p < 0.05; **p < 0.01; ***p < 0.001. (B) Relative LDH activity released by pLVTHM- or anti-TDAG51 shRNA 36-1-infected Ras^V12 ^cells. * p < 0.05.

Since cell growth under anchorage-independent conditions is a balance between cell proliferation and cell death, we sought to evaluate the effect upon cellular death of TDAG51 knock-down. We used an assay of cellular cytotoxicity that measures the release of the lactose deydrogenase enzyme, LDH. LDH release was increased by TDAG51 shRNA-expressing Ras^V12S35 ^cells relative to pLVTHM-infected cells at various time points after the initiation of matrix-detached growth (Figure 8). The difference in LDH release for TDAG51 shRNA-expressing Ras^V12 ^cells was minimal and rarely approached statistical significance. Sub-G1 peaks indicative of dead cells were sometimes seen with cell cycle analysis at late time points, but varied from experiment to experiment. Therefore, for Ras^V12S35^-infected cells, the differences in cell growth after TDAG51 reduction under anchorage-independent conditions resulted from an enhanced rate of cellular proliferation that exceeded a concomitant increase in cell death.

### Reduction of TDAG51 in transformed cells enhances proximal ERK signaling

**Figure 9:**
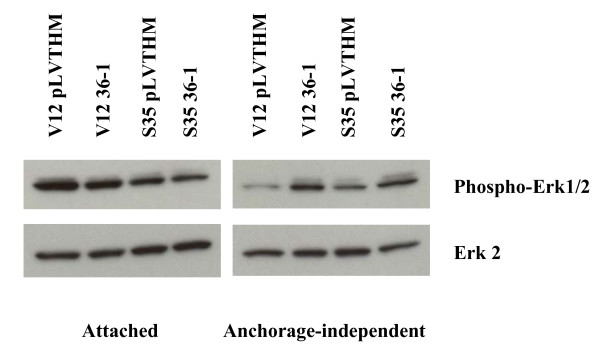
**ERK pathway activation in Ras^V12 ^and Ras^V12S35 ^cells infected with pLVTHM or anti-TDAG51 shRNA 36-1**. Ras^V12 ^or Ras^V12S35 ^cells infected with either pLVTHM vector control or anti-TDAG51 shRNA 36-1 were grown for 72 hours attached to tissue culture plastic (attached) or in ultra-low attachment plates (anchorage-independent) in the presence of 250 ng/mL doxycycline. ERK pathway activation was measured by determination of phosphorylated Erk1/2 levels by western blotting using an anti-phosphorylated Erk mAb (phospho-Erk). The blot was stripped and subsequently probed with an anti-Erk2 mAb (Erk2).

Reducing TDAG51 protein levels in ERK-driven cellular transformation enhanced cell growth under anchorage-independent, but not attached, conditions. To test whether TDAG51 might affect proximal ERK signaling, we examined the activation status of Erk in cells expressing TDAG51-specific shRNA. Interestingly, the levels of phosphorylated Erk were enhanced when TDAG51 protein levels were reduced in Ras^V12S35 ^and Ras^V12 ^cells grown under anchorage-independent, but not attached, conditions (Figure 9). The fact that the activation status of Erk was unchanged in cells grown under attached conditions suggests that reducing TDAG51 expression had no selective effect with regard to ERK activation in these cells. Rather, the enhanced activation of Erk was specific to anchorage-independent growth conditions.

## Discussion

Ras is a common signaling node for various cell surface receptors that contribute to epithelial cell transformation [14]. In this study, we used the hTERT-immortalized human mammary epithelial cell line HME16C to examine which Ras signaling pathways are sufficient for transformation and to identify transcriptional targets downstream of those pathways that might modulate this phenotype. Transduction of HME16C with pathway-discriminating Ras effector domain mutants demonstrated that multiple downstream Ras signal transduction pathways contribute to anchorage-independent growth including Raf-, RalGEF-, and PI3K-mediated signaling. Transformation of HME16C by the Ras^V12G37 ^effector domain mutant but not activated Rlf-CAAX suggest that Ras^V12G37^-binding effectors other than RalGEF contribute to mammary epithelial transformation.

Microarray analyses of Ras^V12 ^and Ras effector domain mutant transduced cells demonstrated a common upregulation of EGFR ligands among transformed cell lines. This suggested that autocrine EGFR ligand secretion was an important component of Ras-mediated cellular transformation. Following blockade of EGFR signaling with the EGFR-specific inhibitor PD153035, the only pathway downstream of Ras that promoted anchorage-independent growth was Raf/ERK, suggesting that Raf activation was able to substitute for EGFR activity in this cell line. In contrast, previous studies with MCF10A cells demonstrated that EGFR tyrosine kinase activity was necessary to inhibit anoikis upon matrix detachment, even in cells expressing activated Raf [16]. By contrast, under matrix detached conditions, the parental HME16C cells are non-proliferative, but do not actively undergo anoikis. The studies here measure a proliferative response of Raf/ERK activation in the absence of a strong apoptotic component. In addition, blockade of MEK activity with the MEK-specific inhibitor PD98059 prevented the transformation of Ras^V12^- and Ras^V12S35^-expressing cells. This, taken together with the finding that Ras^V12^- and Ras^V12S35^-transduced HME16C cells were able to form tumors in nude mice, indicates that the Raf-MEK-Erk axis plays a crucial role in mediating transformation and tumorigenesis in this model. However, it should be noted that Ras-transformed HME16C, as well as the similar HMLER [32], give rise to squamous metaplasias and not adenocarcinomas. It has been hypothesized that HMLE human mammary epithelial cells represent a distinct precursor population from those mammary epithelial cells that give rise to glandular adenocarcinomas, the predominant form of breast cancer [32].

The significantly reduced tumorigenicity of Ras^V12S35^- as compared to Ras^V12^- expressing cells emphasizes the importance of cooperation between Ras signal transduction pathways for a fully transformed phenotype. Following EGFR inhibition, Ras^V12C40^- and Ras^V12G37^- transduced lines lost anchorage-independent growth, suggesting that non-ERK signaling pathways contribute to cellular transformation through an EGFR dependent mechanism. Indeed, one consequence of EGFR activation is ERK pathway activation and may explain the role of EGFR in cooperating with Ras^V12G37^- and Ras^V12C40^-mediated cell signaling to promote anchorage-independent growth.

Microarray analyses identified up-regulation of the PHLDA1 gene product as being correlated with ERK-mediated cellular transformation. Likewise, the protein product of the PHLDA1 gene, TDAG51, displayed an identical expression pattern. Expression of PHLDA1 mRNA and TDAG51 protein in breast cancer has recently been described [17]. Using TDAG51 immunohistochemistry of tissue microarrays, 699 individual primary invasive breast tumor specimens were examined; loss of TDAG51 was found to correlate with a poorer disease-free and overall survival rate in multivariate analysis. Similar results have been found in a small series of melanomas, where TDAG51 immunoreactivity was found to decrease during the progression of melanocytic nevi to primary melanomas and finally to metastatic melanoma [18]. These studies suggest that TDAG51 has a suppressive effect on tumor progression and prompted us to evaluate the cell biological function of TDAG51 in the HME16C transformation model.

The PHLDA1 gene is a member of the pleckstrin homology-related domain family that includes Ipl/Tssc and Tih [33]. PHDLA1 has been described as an immediate early gene with transcriptional activation resulting from engagement of receptors such as the FGF and IGF tyrosine kinase receptors and the T-cell receptor [34-36]. Strong TDAG51 induction by Ras^V12S35 ^transformation of mammary epithelial cells suggests that ERK activation may at least partially explain TDAG induction in these previous reports. *In vitro *studies have suggested a role for TDAG51 in the control of cellular proliferation and in the induction of apoptosis in response to a variety of stresses including proteotoxic cellular stresses such as lung cancer cell responses to chemotherapy[35,37-39].

Overexpression of TDAG51 has been shown to reduce cell proliferation and induce cell death in a variety of cell types including T cells, neuronal, endothelial, melanoma, and cervical carcinoma cell types [34,35,38]. By contrast, TDAG51 functioned as an anti-apoptotic factor downstream of insulin-like growth factor receptor (IGFR) signaling as TDAG51 was required to protect NIH3T3 cells from apoptosis upon IGF-1 withdrawal [36].

Reduction of TDAG51 levels in Ras^V12 ^and Ras^V12S35 ^cells enhanced cell proliferation under anchorage-independent conditions. This suggests that TDAG51 expression limits cellular proliferation. In addition to enhancing cellular proliferation, reduction of TDAG51 protein levels also increased the absolute amount of cell death in transformed Ras^V12S35 ^cells under these same conditions. Therefore, TDAG51 loss, in the context of oncogene activation, may indirectly promote cell death as a consequence of enhanced cell cycling. However, the overall increased cell numbers in anchorage-independent conditions showed that enhanced cellular proliferation exceeded enhanced cell death.

Interestingly, a decrease in TDAG51 expression during Ras-mediated cellular transformation promoted the growth of cells under anchorage-independent conditions but did not affect the growth of attached cells. This suggests that TDAG51 might act in conjunction with both cellular stress, in this case matrix detachment, and a proliferative signal, in this case oncogenic activation. Other studies also have implicated TDAG51 in functioning during cellular stress and survival, particularly endoplasmic reticulum stress associated with the unfolded protein response [37,38]. A mechanism of action for TDAG51 is not known. The preliminary finding that TDAG51 binds to proteins involved in protein translation has been used to suggest that TDAG51 may play a role in regulating protein synthesis in response to proteotoxic stress [40].

Reduction of TDAG51 expression resulted in an increase in Erk activation in cells grown under anchorage-independent conditions. How TDAG51 expression might suppress ERK signaling is not known, but appears to represent a negative feedback pathway that directly or indirectly limits ERK activation. This is not likely to be due to an inhibition of ERK protein synthesis by TDAG51, since Erk2 levels were unaffected by reduced TDAG51. However, another important component of ERK activation is the dual specific ERK phosphatases, a highly regulated class of proteins, whose relative level of activity may be affected by TDAG51 protein levels. Understanding the mechanisms by which TDAG51 regulates ERK pathway activation and the balance between cellular proliferation and apoptosis of transformed cells represents a future challenge. Finally, TDAG51 acts in a suppressive manner during matrix-detached growth of HME16C cells. Taken together with the identification of TDAG51 as a stress-induced gene in a variety of cell lines and a growth inhibitor in melanoma cell lines, it is reasonable to suggest that loss of TDAG51 may act to promote progression of breast cancer through an intrinsic growth regulatory mechanism.

## Conclusion

Expression of activated Ras effector domain mutants that bind Raf, PI3K, or RalGEF are sufficient to induce the anchorage-independent growth of the human mammary epithelial cell line HME16C and are associated with up-regulation of EGFR ligands. However, only the ERK pathway is capable of supporting transformation in the absence of EGFR signaling and of supporting tumorigenesis in nude mice. Up-regulation of TDAG51 occurs during Ras-mediated transformation in an ERK-dependent fashion, but opposes ERK-mediated transformation by suppressing ERK signaling and decreasing cellular proliferation under matrix-detached conditions. Therefore, in this model of mammary epithelial cell transformation, TDAG51 acts as a growth inhibitor of ERK-driven proliferation and may help explain why loss of TDAG51 expression has been found to correlate with progression in human breast cancer and melanoma.

## Abbreviations

EGFR: epidermal growth factor receptor; ErbB2: epidermal growth factor receptor family member B2; Erk: extracellular regulated kinase; PHDLA1: pleckstrin homology domain-like, family A, member 1; PI3K: phsphatidyl inositol 3-kinase; PIK3CA: phosphatidyl inositol 3-kinase, catalytic, alpha polypeptide; PTEN: phosphatase and tensin homologue deleted from chromosome 10; Ral: ras-like gene; RalGEF: Ral guanidine nucleotide exchange factor; Rlf: RalGEF-like factor; shRNA: small hairpin ribonucleic acid; TDAG51: T-cell death associated gene of 51 kDa.

## Competing interests

The authors declare that they have no competing interests. The final text was reviewed and approved by all the listed co-authors. They all contributed to the intellectual content and to the experimental work in an honest manner. The contents have not been published by or submitted to any other journal. No part of this text has been included in any other paper.

## Authors' contributions

Conception and design: MDO and KK. Provision of study materials: MDO, JJY, YW, and KK. Collection and assembly of data: MDO, SJB, and LZ. Data analysis and interpretation: MDO and KK. Manuscript writing: MDO and KK. Final approval of manuscript: MDO, SJB, LZ, JJY, YW, and KK.

## Pre-publication history

The pre-publication history for this paper can be accessed here:



## Supplementary Material

Additional file 1 **Selected microarray data for HME16C cell lines**. The fold up-regulation or down-regulation of genes identified in microarray analysis is indicated for genes where this value exceeded two-fold, organized into categories according to broadly defined gene functions. PHLDA1 is categorized as an up-regulated gene under "Miscellaneous." Click here for file

Additional file 2 **Real-time RT-PCR confirmation of selected microarray data**. Results are for transcripts identified as up- or down-regulated in microarray analysis of cDNA from Ras^V12^-, Ras^V12G37^-, Ras^V12S35^-, Ras^V12C40^-, and Rlf-CAAX-infected HME16C. Numbers represent fold expression changes for transcript levels relative to vector-infected pLRT control cells. Parenthetical numbers indicate mean fold expression changes from cDNA microarray analysis (see Additional file above). Sequences of primers used for real-time RT-PCR analysis are as follows: amphiregulin Forward 5'-CGCCGGTGGTGCTGTCGCTCTT-3' Reverse 5'-TCACTCACAGGGGAAATCTCACTC-3', anterior gradient 2 Forward 5'-GGGGTGACCAACTCATCTGGACTCAG-3' Reverse 5'-GACATACTGGCCATCAGGAGAAAGGTGT-3', CD24 Forward 5'-GCTCCTACCCACGCAGATTTATT-3' Reverse 5'-CACGAAGAGACTGGCTGTTGACT-3', c-met Forward 5'-AAATGGCCACGGGACAACACAA-3' Reverse 5'-TGGGCTGGGGTATAACATTCAAGA-3', CTGF Forward 5'-CTGCCCGGGAAATGCTGCGAGGAGT-3' Reverse 5'-CTGCAGGAGGCGTTGTCATTGGTAA-3', CYR61 Forward 5'-CGGCCCAAGTACTGCGGTTCCT-3' Reverse 5'-ATTGGCATGCGGGCAGTTGTAGTT-3', Decorin Forward 5'-ACTTCTGCCCACCTGGACACAACA-3' Reverse 5'-ATGGCAGAGCGCACGTAGACACA-3', DICER-1 Forward 5'-CAGGAAATACCCGTGCAACCAACTA-3' Reverse 5'-GCATTACGGCCATCACAGGACTTC-3', E-cadherin Forward 5'-GGTATCTTCCCCGCCCTGCCAATCC-3' Reverse 5'-AACCGCTTCCTTCATAGTCAAACACGAG-3', EphA2 Forward 5'-CCCCTTCCGCCCCACACTACCTCACAGC-3' Reverse 5'-ACACGGCCCGCATTCCCCAGACTCG-3', Epiregulin Forward 5'-TTGTATTTTTAGTAGAGGCGGGGTTTCA-3' Reverse 5'-TCGGGCACAGATGTTCAAGTCAC-3', ETS-1 Forward 5'-ACTCGGGGGCCAGGACTCTTTTGAA-3' Reverse 5'-CACGGTCCCGCACATAGTCCTTGAA-3', ETS variant gene 5 Forward 5'-TCGGGGACGTCTACGGTTTCTACT-3' Reverse 5'-AAGACTGTAAACGGCTACCATTGA-3', FAT-2 Forward 5'-GCTGGACATCAAACGGGCTAACAT-3' Reverse 5'-ACCGCATCTGAACCCCCACTGAAT-3', HMGA-1 Forward 5'-GCTCACCCTGCCCGCTCCCAACC-3' Reverse 5'-GCCCCAGCCCCTCTTCCCCACAAA-3', HMGA-2 Forward 5'-CTGATAAGCAAGAGTGGGCGGGTGAGAA-3' Reverse 5'-ACAGGGAGTGGGTTGGGGTGGTATTTGA-3', Secreted frizzled-related protein 1 Forward 5'-CCCGCTCCCTTTCCCTCCATA-3' Reverse 5'-GCTCTCACTTTCCGCCCAATCC-3', Tenascin C Forward 5'-GCGGCCCAGAGCGAGGAA-3' Reverse 5'-TATTGCGATGTTGTCACTGGGAGATTTT-3', TGF-a Forward 5'-CCGCCTTGGTGGTGGTCTCC-3' Reverse 5'-AGGGCGCTGGGCTTCTCGTG-3', TGF-β2 Forward 5'-CCCGCCCACTTTCTACAGACCCTACT-3' Reverse 5'-CATTCGCCTTCTGCTCTTGTTTTCAC-3', TGF-β receptor 2 Forward 5'-ACAGTGGCAGTCAAGATCTTTCCCTATG-3' Reverse 5'-CAACTCCGTCTTCCGCTCCTCAG-3', TRIM29 Forward 5'-ATGCATGCGCCACGTTGAGAAGAT-3' Reverse 5'-GGTGAAGCGGCCAGGAGACGAG-3'. Click here for file
