# Supplementary material for: TDAG51 is an ERK signaling target that opposes ERK-mediated HME16C mammary epithelial cell transformation
Source: BMC Cancer. 2008 Jul 2;8:189. doi: 10.1186/1471-2407-8-189 (PMC2474852; doi:10.1186/1471-2407-8-189)
Supplement: Additional file 1 — Selected microarray data for HME16C cell lines. The fold up-regulation or down-regulation of genes identified in microarray analysis is indicated for genes where this value exceeded two-fold, organized into categories according to broadly defined gene functions. PHLDA1 is categorized as an up-regulated gene under "Miscellaneous." [file 1471-2407-8-189-S1.doc]

|  |  |  |  |  | **Cell Adhesion/ECM/ECM proteolysis** |
| --- | --- | --- | --- | --- | --- |
|  |  |  |  |  |  |
|  |  |  |  |  | **Up-regulated genes** |
| **V12** | **S35** | **G37** | **C40** | **Gene** | **Description** |
| 4.2 | 2.4 | 1.9 | 2.7 | ADAMTS1 | a disintegrin-like and metalloprotease (reprolysin type) with thrombospondin type 1 motif, 1 |
| 3.9 | 0.7 | 2.5 | 1.0 | ITGB5 | integrin, beta 5 (ITGB5), mRNA. |
| 3.4 | 3.8 | 1.0 | 2.7 | GPC6 | Glypican 6 |
| 3.1 | 1.0 | 3.1 | 1.0 | TNC | tenascin C (hexabrachion) (TNC), mRNA. |
| 3.1 | 1.0 | 1.1 | 1.2 | CDH11 | cadherin 11, type 2, OB-cadherin (osteoblast) (CDH11), transcript variant 1, mRNA. |
| 3.1 | 3.0 | 1.6 | 1.7 | DCBLD2 | discoidin, CUB and LCCL domain containing 2 (DCBLD2), mRNA. |
| 3.1 | 2.6 | 1.9 | 1.7 | PLAUR | plasminogen activator, urokinase receptor (PLAUR), transcript variant 1, mRNA. |
| 2.5 | 1.0 | 1.0 | 1.0 | JAM3 | junctional adhesion molecule 3 (JAM3), mRNA. |
| 2.3 | 2.5 | 1.4 | 1.4 | LAMC2 | laminin, gamma 2 (LAMC2), transcript variant 1, mRNA. |
| 2.3 | 1.7 | 1.5 | 1.5 | CDSN | corneodesmosin (CDSN), mRNA. |
| 2.2 | 0.8 | 1.3 | 1.1 | ITGB1 | Integrin, beta 1 (fibronectin receptor, beta polypeptide, antigen CD29 includes MDF2, MSK12) |
|  |  |  |  |  |  |
|  |  |  |  |  | **Down-regulated genes** |
| **V12** | **S35** | **G37** | **C40** | **Gene** | **Description** |
| 11.1 | 4.3 | 2.4 | 2.2 | FAT2 | FAT tumor suppressor homolog 2 (Drosophila) (FAT2), mRNA. |
| 9 |  | 3.9 |  | TIMP3 | tissue inhibitor of metalloproteinase 3 (Sorsby fundus dystrophy, pseudoinflammatory) (TIMP3) |
| 5.1 | 3 | 2.5 | 2.6 | COL7A1 | collagen, type VII, alpha 1 (epidermolysis bullosa, dystrophic, dominant and recessive) (COL7A1) |
| 4.2 | 2.1 | 1.8 | 1.8 | LAMA5 | laminin, alpha 5 (LAMA5), mRNA. |
| 3.4 | 2 |  | 1.7 | CEACAM21 | carcinoembryonic antigen-related cell adhesion molecule 21 (CEACAM21), mRNA. |
| 3.1 |  | 1.3 |  | COL5A1 | collagen, type V, alpha 1 (COL5A1), mRNA. |
| 3.1 | 2 | 1.8 | 1.6 | CDH3 | cadherin 3, type 1, P-cadherin (placental) (CDH3), mRNA. |
| 2.9 |  | 1.7 |  | HSPG2 | heparan sulfate proteoglycan 2 (perlecan) (HSPG2), mRNA. |
| 2.8 | 1.9 | 2.5 | 1.8 | CELSR2 | cadherin, EGF LAG seven-pass G-type receptor 2 (flamingo homolog, Drosophila) (CELSR2), mRNA. |
| 2.5 | 2.1 | 2.2 | 1.9 | JUP | junction plakoglobin (JUP), transcript variant 2, mRNA. |
| 2.5 | 1.9 | 1.8 | 1.5 | PKP3 | plakophilin 3 (PKP3), mRNA. |
| 2.5 | 2.6 |  | 1.6 | CTHRC1 | collagen triple helix repeat containing 1 (CTHRC1), mRNA. |
| 2.4 | 1.5 | 1.8 | 1.6 | DSC3 | desmocollin 3 (DSC3), transcript variant Dsc3a, mRNA. |
| 2.3 | 1.6 | 1.1 | 1.2 | EFEMP1 | EGF-containing fibulin-like extracellular matrix protein 1 (EFEMP1), transcript variant 2, mRNA. |
| 2.2 | 1.7 | 2 | 1.6 | COL4A5 | collagen, type IV, alpha 5 (Alport syndrome) (COL4A5), transcript variant 1, mRNA. |
| 2.2 | 1 | 1.6 | 1.2 | LAD1 | ladinin 1 (LAD1), mRNA. |
|  |  |  |  |  |  |
|  |  |  |  |  |  |
|  |  |  |  |  | **Cell Signaling** |
|  |  |  |  |  |  |
|  |  |  |  |  | **Up-regulated genes** |
| **V12** | **S35** | **G37** | **C40** | **Gene** | **Description** |
| 25.2 | 23.3 | 28.5 | 12.7 | HRAS | v-Ha-ras Harvey rat sarcoma viral oncogene homolog (HRAS), transcript variant 1, mRNA. |
| 10.4 | 5.5 | 3.8 | 2.3 | DUSP6 | dual specificity phosphatase 6 (DUSP6), transcript variant 2, mRNA. |
| 7.5 | 4.8 | 2.6 | 2.3 | DUSP5 | dual specificity phosphatase 5 (DUSP5), mRNA. |
| 6.5 | 4.1 | 1.5 | 1.3 | TRIB1 | tribbles homolog 1 (Drosophila) (TRIB1), mRNA. |
| 4.8 | 2.9 | 1.8 | 1.7 | DUSP4 | dual specificity phosphatase 4 (DUSP4), transcript variant 1, mRNA. |
| 4.7 | 1.0 | 1.9 | 1.0 | DOCK4 | Dedicator of cytokinesis 4 |
| 4.7 | 2.3 | 1.4 | 1.4 | ERRFI1 | ERBB receptor feedback inhibitor 1 (ERRFI1), mRNA. |
| 4.1 | 2.5 | 1.6 | 1.4 | CCND1 | cyclin D1 (PRAD1: parathyroid adenomatosis 1) (CCND1), mRNA. |
| 3.8 | 1.9 | 1.7 | 2.0 | EDG2 | endothelial differentiation, lysophosphatidic acid G-protein-coupled receptor, 2 (EDG2) |
| 3.8 | 2.8 | 2.1 | 2.1 | SPHK1 | sphingosine kinase 1 (SPHK1), mRNA. |
| 2.8 | 1.0 | 1.6 | 1.0 | RIPK2 | receptor-interacting serine-threonine kinase 2 (RIPK2), mRNA. |
| 2.8 | 1.0 | 1.2 | 1.0 | BCL10 | B-cell CLL/lymphoma 10 (BCL10), mRNA. |
| 2.7 | 1.6 | 1.0 | 1.3 | STK10 | serine/threonine kinase 10 (STK10), mRNA. |
| 2.7 | 1.4 | 1.0 | 1.1 | CARD11 | caspase recruitment domain family, member 11 (CARD11), mRNA. |
| 2.6 | 1.0 | 1.4 | 0.9 | GTPBP4 | GTP binding protein 4 (GTPBP4), mRNA. |
| 2.6 | 2.9 | 1.9 | 1.8 | TM4SF9 | Transmembrane 4 superfamily member 9 |
| 2.5 | 1.7 | 1.1 | 1.0 | BCAR3 | breast cancer anti-estrogen resistance 3 (BCAR3), mRNA. |
| 2.5 | 1.5 | 1.0 | 1.2 | DUSP10 | dual specificity phosphatase 10 (DUSP10), transcript variant 3, mRNA. |
| 2.5 | 2.3 | 1.2 | 1.6 | ABL2 | V-abl Abelson murine leukemia viral oncogene homolog 2 (arg, Abelson-related gene) |
| 2.4 | 1.5 | 1.1 | 1.1 | PCTK2 | PCTAIRE protein kinase 2 (PCTK2), mRNA. |
| 2.4 | 1.6 | 1.2 | 1.4 | EPS8 | epidermal growth factor receptor pathway substrate 8 (EPS8), mRNA. |
| 2.4 | 1.5 | 1.5 | 1.4 | PPAP2B | phosphatidic acid phosphatase type 2B (PPAP2B), transcript variant 2, mRNA. |
| 2.3 | 2.0 | 1.1 | 1.4 | PLCE1 | phospholipase C, epsilon 1 (PLCE1), mRNA. |
| 2.3 | 0.9 | 1.3 | 1.0 | CaMKIINalpha | Calcium/calmodulin-dependent protein kinase II |
| 2.2 | 2.5 | 1.6 | 2.1 | PAK2 | P21 (CDKN1A)-activated kinase 2 |
| 2.2 | 2.1 | 1.3 | 1.7 | RAP1B | RAP1B, member of RAS oncogene family (RAP1B), mRNA. |
| 2.2 | 1.6 | 1.1 | 1.3 | HHIP | hedgehog interacting protein (HHIP), mRNA. |
| 2.2 | 1.2 | 1.2 | 1.1 | MAP4K4 | mitogen-activated protein kinase kinase kinase kinase 4 (MAP4K4), transcript variant 1, mRNA. |
| 2.2 | 1.5 | 1.3 | 1.3 | PAK1IP1 | PAK1 interacting protein 1 (PAK1IP1), mRNA. |
| 2.2 | 1.7 | 1.5 | 1.8 | RALA | V-ral simian leukemia viral oncogene homolog A (ras related) |
| 2.1 | 1.4 | 1.2 | 1.3 | ANXA3 | annexin A3 (ANXA3), mRNA. |
| 2.1 | 1.6 | 1.2 | 1.4 | FADD | Fas (TNFRSF6)-associated via death domain (FADD), mRNA. |
| 2.0 | 1.7 | 1.0 | 1.2 | PPP1R3B | protein phosphatase 1, regulatory (inhibitor) subunit 3B (PPP1R3B), mRNA. |
|  |  |  |  |  |  |
|  |  |  |  |  | **Down-regulated genes** |
| **V12** | **S35** | **G37** | **C40** | **Gene** | **Description** |
| 16.8 | 4.8 |  | 5.4 | RIPK3 | receptor-interacting serine-threonine kinase 3 (RIPK3), mRNA. |
| 4.5 | 2.9 | 2.2 | 1.3 | DCAMKL1 | doublecortin and CaM kinase-like 1 (DCAMKL1), mRNA. |
| 4.1 |  | 1.3 |  | STK6 | serine/threonine kinase 6 (STK6), transcript variant 6, mRNA. |
| 3.9 | 1.5 | 1.8 |  | CDC25B | Cell division cycle 25B |
| 3 | 2 | 1.9 | 1.8 | SFRP1 | secreted frizzled-related protein 1 (SFRP1), mRNA. |
| 2.8 | 1.8 | 1.5 | 1.9 | RIN2 | Ras and Rab interactor 2 (RIN2), mRNA. |
| 2.6 | 3.3 |  | 2.3 | FGFBP1 | fibroblast growth factor binding protein 1 (FGFBP1), mRNA. |
| 2.6 | 2 | 1.9 | 1.6 | RIPK4 | receptor-interacting serine-threonine kinase 4 (RIPK4), mRNA. |
| 2.5 |  | 1 |  | UCN | urocortin (UCN), mRNA. |
| 2.5 |  | 1.2 |  | MKNK2 | MAP kinase interacting serine/threonine kinase 2 (MKNK2), mRNA. |
| 2.5 | 1.8 | 1.5 | 1.5 | DDR1 | discoidin domain receptor family, member 1 (DDR1), transcript variant 2, mRNA. |
| 2.4 | 1.3 | 1.2 | 1.1 | BDKRB2 | bradykinin receptor B2 (BDKRB2), mRNA. |
| 2.3 | 1.7 | 1.9 | 1.5 | JAG2 | jagged 2 (JAG2), transcript variant 2, mRNA. |
| 2.1 | 1.5 | 1.4 | 1.4 | SHC1 | SHC (Src homology 2 domain containing) transforming protein 1 (SHC1), transcript variant 2, mRNA. |
| 2.1 | 1.4 | 1.4 | 1.1 | PTPNS1 | protein tyrosine phosphatase, non-receptor type substrate 1 (PTPNS1), mRNA. |
|  |  |  |  |  |  |
|  |  |  |  |  |  |
|  |  |  |  |  | **Cytoskeleton/Cytoskeletal remodeling/Cell Junctions** |
|  |  |  |  |  |  |
|  |  |  |  |  | **Up-regulated genes** |
| **V12** | **S35** | **G37** | **C40** | **Gene** | **Description** |
| 4.9 | 3.0 | 1.4 | 1.7 | RND3 | Rho family GTPase 3 (RND3), mRNA. |
| 3.0 | 2.0 | 2.3 | 2.1 | CDC42EP2 | CDC42 effector protein (Rho GTPase binding) 2 (CDC42EP2), mRNA. |
| 2.9 | 2.6 | 1.6 | 1.9 | EPLIN | epithelial protein lost in neoplasm beta (EPLIN), mRNA. |
| 2.6 | 1.3 | 1.3 | 1.0 | STARD13 | START domain containing 13 (STARD13), transcript variant gamma, mRNA. |
| 2.6 | 2.0 | 1.1 | 1.4 | ARPC3 | actin related protein 2/3 complex, subunit 3, 21kDa (ARPC3), mRNA. |
| 2.4 | 1.9 | 1.3 | 1.6 | CORO1C | coronin, actin binding protein, 1C (CORO1C), mRNA. |
| 2.3 | 1.6 | 1.4 | 1.3 | RGNEF | PREDICTED: Homo sapiens Rho-guanine nucleotide exchange factor (RGNEF), mRNA. |
| 2.2 | 1.0 | 1.1 | 1.0 | DST | Dystonin |
| 2.1 | 1.5 | 1.7 | 1.8 | TMSB4X | Thymosin, beta 4, X-linked |
| 2.1 | 1.4 | 1.0 | 0.9 | PLEK2 | pleckstrin 2 (PLEK2), mRNA. |
| 2.0 | 1.9 | 1.3 | 1.4 | WASL | Wiskott-Aldrich syndrome-like (WASL), mRNA. |
|  |  |  |  |  |  |
|  |  |  |  |  | **Down-regulated genes** |
| **V12** | **S35** | **G37** | **C40** | **Gene** | **Description** |
| 38.7 | 28.2 | 10.2 | 11.7 | KRT15 | keratin 15 (KRT15), mRNA. |
| 7.6 | 3 | 3.5 | 3.1 | DSG3 | desmoglein 3 (pemphigus vulgaris antigen) (DSG3), mRNA. |
| 5.8 | 4.5 | 2.3 | 1.8 | KRT12 | keratin 12 (Meesmann corneal dystrophy) (KRT12), mRNA. |
| 4.8 | 2.4 | 1.2 | 1.2 | KRT17 | keratin 17 (KRT17), mRNA. |
| 3.6 |  | 1.3 |  | ADD3 | adducin 3 (gamma) (ADD3), transcript variant 2, mRNA. |
| 3 | 1.8 | 1.6 | 1.4 | LIMK2 | LIM domain kinase 2 (LIMK2), transcript variant 2a, mRNA. |
| 2.9 |  | 1.5 |  | GSN | gelsolin (amyloidosis, Finnish type) (GSN), transcript variant 2, mRNA. |
| 2.7 | 1.5 | 2.1 | 2.1 | ARHGEF4 | Rho guanine nucleotide exchange factor (GEF) 4 (ARHGEF4), transcript variant 1, mRNA. |
| 2.5 | 1.2 | 1.3 | 1.1 | MYH7 | myosin, heavy polypeptide 7, cardiac muscle, beta (MYH7), mRNA. |
| 2.3 | 1.3 | 1.6 | 1.3 | EPS8L2 | EPS8-like 2 (EPS8L2), mRNA. |
| 2.2 | 1.7 | 1.5 | 1.3 | KRT2A | keratin 2A (epidermal ichthyosis bullosa of Siemens) (KRT2A), mRNA. |
| 2.2 | 2.1 |  | 1.4 | ARPC1A | Actin related protein 2/3 complex, subunit 1A, 41kDa |
|  |  |  |  |  |  |
|  |  |  |  |  |  |
|  |  |  |  |  | **Growth factor receptors/GFR ligands/angiogenesis** |
|  |  |  |  |  |  |
|  |  |  |  |  | **Up-regulated genes** |
| **V12** | **S35** | **G37** | **C40** | **Gene** | **Description** |
| 7.2 | 4.3 | 1.0 | -1.1 | BMP2 | bone morphogenetic protein 2 (BMP2), mRNA. |
| 7.0 | 1.0 | 2.4 | 1.3 | EREG | epiregulin (EREG), mRNA. |
| 5.9 | 4.2 | 3.6 | 3.1 | CTGF | connective tissue growth factor (CTGF), mRNA. |
| 4.2 | 4.1 | 2.2 | 2.1 | EPHA2 | EPH receptor A2 (EPHA2), mRNA. |
| 4.1 | 4.4 | 1.0 | 1.9 | CSF3 | colony stimulating factor 3 (granulocyte) (CSF3), transcript variant 2, mRNA. |
| 3.8 | -1.3 | 1.3 | 1.0 | IL1B | interleukin 1, beta (IL1B), mRNA. |
| 3.4 | 3.1 | 2.1 | 2.4 | VEGFC | vascular endothelial growth factor C (VEGFC), mRNA. |
| 3.4 | 1.3 | -1.4 | -1.1 | TGFA | transforming growth factor, alpha (TGFA), mRNA. |
| 3.3 | -1.3 | 1.2 | 1.4 | FZD8 | frizzled homolog 8 (Drosophila) (FZD8), mRNA. |
| 3.3 | 4.0 | 1.6 | 1.6 | NRP1 | neuropilin 1 (NRP1), mRNA. |
| 3.2 | 3.1 | 1.8 | 2.3 | DKK1 | dickkopf homolog 1 (Xenopus laevis) (DKK1), mRNA. |
| 3.1 | 2.3 | 1.6 | 1.4 | MET | met proto-oncogene (hepatocyte growth factor receptor) (MET), mRNA. |
| 3.0 | 1.0 | 2.3 | 1.0 | CKLFSF7 | chemokine-like factor super family 7 (CKLFSF7), transcript variant 2, mRNA. |
| 3.0 | 1.0 | 1.4 | -1.1 | TNFRSF10D | tumor necrosis factor receptor superfamily, member 10d, decoy with truncated death domain |
| 2.5 | 2.6 | 2.6 | 2.1 | CYR61 | cysteine-rich, angiogenic inducer, 61 (CYR61), mRNA. |
| 2.5 | 1.0 | 2.1 | 1.0 | PBEF1 | pre-B-cell colony enhancing factor 1 (PBEF1), transcript variant 1, mRNA. |
| 2.4 | 1.0 | 1.7 | 1.0 | AREG | amphiregulin (schwannoma-derived growth factor) (AREG), mRNA. |
|  |  |  |  |  |  |
|  |  |  |  |  | **Down-regulated genes** |
| **V12** | **S35** | **G37** | **C40** | **Gene** | **Description** |
| 5.5 |  | 2.3 | 1.2 | IL1F9 | interleukin 1 family, member 9 (IL1F9), mRNA. |
| 5.4 | 2.2 | 3.4 | 2.2 | GPR56 | G protein-coupled receptor 56 (GPR56), transcript variant 3, mRNA. |
| 4.8 | 2.7 |  | 2.9 | RET | Ret proto-oncogene (multiple endocrine neoplasia and medullary thyroid carcinoma 1) |
| 4.2 | 1.7 |  | 1.2 | SSTR1 | somatostatin receptor 1 (SSTR1), mRNA. |
| 3.8 |  | 1.1 |  | NOTCH1 | Notch homolog 1, translocation-associated (Drosophila) (NOTCH1), mRNA. |
| 3.4 |  | 1.7 |  | PLXNB1 | plexin B1 (PLXNB1), mRNA. |
| 3.4 | 1.8 | 2 | 1 | ARTN | artemin (ARTN), transcript variant 3, mRNA. |
| 3.1 | 1.8 |  | 1.5 | CCR9 | chemokine (C-C motif) receptor 9 (CCR9), transcript variant B, mRNA. |
| 3.1 | 1.9 |  | 1.4 | TRBV24-1 | T cell receptor beta variable 24-1 |
| 2.3 | 1.3 | 1.3 | 1 | EFNA1 | ephrin-A1 (EFNA1), transcript variant 2, mRNA. |
| 2.1 | 1.4 | 1.6 | 1.5 | IL17 | interleukin 17 (cytotoxic T-lymphocyte-associated serine esterase 8) (IL17), mRNA. |
| 2.1 | 1.7 | 1.3 | 1.4 | EPO | erythropoietin (EPO), mRNA. |
|  |  |  |  |  |  |
|  |  |  |  |  |  |
|  |  |  |  |  | **Metabolism** |
|  |  |  |  |  |  |
|  |  |  |  |  | **Up-regulated genes** |
| **V12** | **S35** | **G37** | **C40** | **Gene** | **Description** |
| 7.5 | 3.3 | 2.0 | 2.4 | AOX1 | aldehyde oxidase 1 (AOX1), mRNA. |
| 4.6 | 2.7 | 1.7 | 1.6 | GSTO1 | glutathione S-transferase omega 1 (GSTO1), mRNA. |
| 4.5 | 2.9 | 1.9 | 2.0 | ODC1 | ornithine decarboxylase 1 (ODC1), mRNA. |
| 3.4 | 2.2 | 1.3 | 1.4 | IDS | iduronate 2-sulfatase (Hunter syndrome) (IDS), transcript variant 1, mRNA. |
| 3.3 | 2.0 | 1.7 | 1.2 | ENTPD7 | ectonucleoside triphosphate diphosphohydrolase 7 (ENTPD7), mRNA. |
| 3.1 |  |  |  | AADACL1 | arylacetamide deacetylase-like 1 (AADACL1), mRNA. |
| 3.0 | 1.7 | 1.5 | 1.3 | NP | nucleoside phosphorylase (NP), mRNA. |
| 2.9 | 2.4 | 1.3 | 1.9 | UPP1 | uridine phosphorylase 1 (UPP1), transcript variant 1, mRNA. |
| 2.9 | 3.1 | 1.9 | 2.4 | UAP1 | UDP-N-acteylglucosamine pyrophosphorylase 1 (UAP1), mRNA. |
| 2.7 | 2.1 | 1.4 | 1.6 | SOAT1 | sterol O-acyltransferase (acyl-Coenzyme A: cholesterol acyltransferase) 1 (SOAT1) |
| 2.7 | 1.0 | 1.4 | 1.0 | MFTC | mitochondrial folate transporter/carrier (MFTC), mRNA. |
| 2.7 | 2.3 | 1.6 | 2.0 | UGCG | UDP-glucose ceramide glucosyltransferase |
| 2.6 | 2.4 | 1.8 | 2.1 | B3GNT1 | UDP-GlcNAc:betaGal beta-1,3-N-acetylglucosaminyltransferase 1 (B3GNT1), transcript variant 2 |
| 2.6 | 2.1 | 1.2 | 1.2 | AMMECR1 | Hypothetical protein LOC286505 |
| 2.5 | 0.7 | 1.3 | 1.1 | RFK | riboflavin kinase (RFK), mRNA. |
| 2.5 | 1.0 | 1.2 | 1.0 | 2'-PDE | 2'-phosphodiesterase |
| 2.5 | 2.1 | 1.7 | 1.7 | PPM2C | protein phosphatase 2C, magnesium-dependent, catalytic subunit (PPM2C) |
| 2.4 | 1.6 | 1.2 | 1.2 | CHST2 | carbohydrate (N-acetylglucosamine-6-O) sulfotransferase 2 (CHST2), mRNA. |
| 2.4 | 1.0 | 1.2 | 1.0 | HK2 | hexokinase 2 (HK2), mRNA. |
| 2.3 | 2.1 | 1.6 | 1.7 | SAT | spermidine/spermine N1-acetyltransferase (SAT), mRNA. |
| 2.3 | 1.6 | 1.4 | 1.7 | GBE1 | glucan (1,4-alpha-), branching enzyme 1 (glycogen branching enzyme, GBE1), mRNA. |
| 2.3 | 1.3 | 1.1 | 1.1 | GALNACT-2 | chondroitin sulfate GalNAcT-2 (GALNACT-2), mRNA. |
| 2.3 | 1.4 | 1.5 | 1.4 | EPAS1 | endothelial PAS domain protein 1 (EPAS1), mRNA. |
| 2.3 | 1.5 | 1.5 | 1.4 | GYG | glycogenin (GYG), mRNA. |
| 2.3 | 2.0 | 1.2 | 1.3 | MGLL | monoglyceride lipase (MGLL), transcript variant 1, mRNA. |
| 2.3 | 1.1 | 1.7 | 1.2 | GLRX | glutaredoxin (thioltransferase) (GLRX), mRNA. |
| 2.2 | 0.8 | 1.3 | 0.9 | MICAL2 | Flavoprotein oxidoreductase MICAL2 |
| 2.0 | 1.0 | 1.3 | 1.1 | HPCL2 | 2-hydroxyphytanoyl-CoA lyase (HPCL2), mRNA. |
|  |  |  |  |  |  |
|  |  |  |  |  | **Down-regulated genes** |
| **V12** | **S35** | **G37** | **C40** | **Gene** | **Description** |
| 8.8 | 5.2 | 5.4 | 4.9 | SULF2 | sulfatase 2 (SULF2), transcript variant 2, mRNA. |
| 7.4 | 3.1 |  | 2.5 | MAOB | monoamine oxidase B (MAOB), nuclear gene encoding mitochondrial protein, mRNA. |
| 5 | 2.8 | 2.4 | 1.6 | ALDH3A2 | aldehyde dehydrogenase 3 family, member A2 (ALDH3A2), mRNA. |
| 4.5 | 3 | 2.3 | 1.9 | GLUL | glutamate-ammonia ligase (glutamine synthase) (GLUL), mRNA. |
| 4.4 | 3.8 | 2.3 | 1.8 | CROT | carnitine O-octanoyltransferase (CROT), mRNA. |
| 4.3 |  | 1.6 |  | EPHX1 | epoxide hydrolase 1, microsomal (xenobiotic) (EPHX1), mRNA. |
| 3.9 | 3.2 | 1.6 | 1.6 | ASS | argininosuccinate synthetase (ASS), transcript variant 1, mRNA. |
| 3.5 | 2.1 | 2 | 1.8 | IMPA2 | inositol(myo)-1(or 4)-monophosphatase 2 (IMPA2), mRNA. |
| 3.5 | 2 | 1.6 | 1.8 | CYP2C18 | cytochrome P450, family 2, subfamily C, polypeptide 18 (CYP2C18), mRNA. |
| 3.3 | 1.8 | 2.7 | 1.6 | CA9 | carbonic anhydrase IX (CA9), mRNA. |
| 3.2 | 1.4 | 1.2 | 1.4 | LTC4S | leukotriene C4 synthase (LTC4S), transcript variant 1, mRNA. |
| 3 | 2.6 | 2.3 | 1.5 | MAOA | monoamine oxidase A (MAOA), nuclear gene encoding mitochondrial protein, mRNA. |
| 2.9 | 1 | 1.7 | 1.2 | DHRS1 | dehydrogenase/reductase (SDR family) member 1 (DHRS1), mRNA. |
| 2.6 | 2 |  | 1.5 | CYP11B2 | cytochrome P450, family 11, subfamily B, polypeptide 2 (CYP11B2) |
| 2.6 | 1.6 | 1.6 | 1.4 | CMAH | Cytidine monophosphate-N-acetylneuraminic acid hydroxylase |
| 2.5 |  | 1 |  | H6PD | Hexose-6-phosphate dehydrogenase (glucose 1-dehydrogenase) |
| 2.5 | 1.3 | 1.2 |  | HDHD2 | haloacid dehalogenase-like hydrolase domain containing 2 (HDHD2), mRNA. |
| 2.5 | 1.7 | 1.2 | 1.4 | MRC2 | Mannose receptor, C type 2 |
| 2.5 | 1.6 | 1.2 | 1.5 | ALDH7A1 | aldehyde dehydrogenase 7 family, member A1 (ALDH7A1), mRNA. |
| 2.4 | 1.9 | 1.5 | 1.6 | CAT | catalase (CAT), mRNA. |
| 2.3 | 1.4 | 1.9 | 1.4 | AK3 | Adenylate kinase 3 |
| 2.3 | 1.5 | 1.2 | 1.4 | CPS1 | carbamoyl-phosphate synthetase 1, mitochondrial (CPS1), mRNA. |
| 2.2 | 1.5 | 1.2 | 1 | IDH1 | isocitrate dehydrogenase 1 (NADP+), soluble (IDH1), mRNA. |
| 2.2 | 1.3 | 1.3 | 1.1 | CA12 | carbonic anhydrase XII (CA12), transcript variant 2, mRNA. |
| 2.2 | 1.3 | 2.7 | 1.6 | ST6GALNAC2 | ST6 (alpha-N-acetyl-neuraminyl-2,3-beta-galactosyl-1, 3)-N-acetylgalactosaminide |
| 2.1 | 1.4 | 1.2 | 1.2 | GAA | glucosidase, alpha; acid (Pompe disease, glycogen storage disease type II) (GAA), mRNA. |
| 2.1 | 1.5 | 1 | 1 | EHMT2 | euchromatic histone-lysine N-methyltransferase 2 (EHMT2), transcript variant NG36/G9a-SPI, mRNA. |
| 2.1 | 1.4 | 1.3 | 1.2 | SHMT1 | serine hydroxymethyltransferase 1 (soluble) (SHMT1), transcript variant 2, mRNA. |
|  |  |  |  |  |  |
|  |  |  |  |  |  |
|  |  |  |  |  | **Miscellaneous** |
|  |  |  |  |  |  |
|  |  |  |  |  | **Up-regulated genes** |
| **V12** | **S35** | **G37** | **C40** | **Gene** | **Description** |
| 22.3 | 9.1 | 1.0 | 2.5 |  | Homo sapiens cDNA FLJ11373 fis, clone HEMBA1000376 |
| 21.6 | 13.8 | 2.9 | 2.5 | PHLDA1 | Pleckstrin homology-like domain, family A, member 1 |
| 9.9 | 1.0 | 2.2 | 1.0 | PTGS2 | prostaglandin-endoperoxide synthase 2 (prostaglandin G/H synthase and cyclooxygenase) (PTGS2) |
| 8.3 | 1.3 | 2.6 | 1.1 | PHLDA1 | Pleckstrin homology-like domain, family A, member 1 |
| 7.9 | 11.5 | 1.0 | 5.4 | DLEU2 | deleted in lymphocytic leukemia, 2 (DLEU2), mRNA. |
| 6.8 | 1.0 | 2.0 | 1.0 | ANTXR2 | anthrax toxin receptor 2 (ANTXR2), mRNA. |
| 6.5 | 3.0 | 1.8 | 1.8 | HSPH1 | heat shock 105kDa/110kDa protein 1 (HSPH1), mRNA. |
| 5.7 | 4.7 | 1.0 | 2.5 | CNGA3 | cyclic nucleotide gated channel alpha 3 (CNGA3), mRNA. |
| 5.5 | 1.0 | 1.6 | 0.8 |  | Homo sapiens, clone IMAGE:3881549, mRNA |
| 5.5 | 3.2 | 2.3 | 1.5 | ANTXR2 | anthrax toxin receptor 2 (ANTXR2), mRNA. |
| 5.4 | 1.0 | 2.8 | 1.0 | LETM2 | leucine zipper-EF-hand containing transmembrane protein 2 (LETM2), mRNA. |
| 4.7 | 2.9 | 1.0 | 2.1 | FLJ23312 | Hypothetical protein FLJ23312 |
| 4.6 | 3.2 | 1.0 | 1.8 | PLXNA4B | plexin A4, B (PLXNA4B), mRNA. |
| 4.3 | 2.3 | 1.4 | 1.3 | CHORDC1 | cysteine and histidine-rich domain (CHORD)-containing, zinc binding protein 1 (CHORDC1), mRNA. |
| 4.3 | 2.3 | 1.4 | 1.3 | NPC1 | Niemann-Pick disease, type C1 (NPC1), mRNA. |
| 4.1 | 2.5 | 0.5 | 1.5 | RAD51L3 | RAD51-like 3 (S. cerevisiae) (RAD51L3), transcript variant 4, mRNA. |
| 3.9 | 0.6 | 1.4 | 0.6 | EIF2C2 | Eukaryotic translation initiation factor 2C, 2 |
| 3.9 | 2.6 | 1.7 | 1.8 | DNAJA1 | DnaJ (Hsp40) homolog, subfamily A, member 1 |
| 3.8 | 1.6 | 2.3 | 1.6 | DAB2 | disabled homolog 2, mitogen-responsive phosphoprotein (Drosophila) (DAB2), mRNA. |
| 3.8 | 1.0 | 1.2 | 1.0 | HSPA1A | heat shock 70kDa protein 1A (HSPA1A), mRNA. |
| 3.7 | 1.0 | 1.1 | 1.0 | HSPA1A | heat shock 70kDa protein 1A (HSPA1A), mRNA. |
| 3.7 | 1.6 | 1.3 | 1.2 | KCNK1 | potassium channel, subfamily K, member 1 (KCNK1), mRNA. |
| 3.6 | 1.0 | 1.3 | 1.0 | HSPA1A | heat shock 70kDa protein 1A (HSPA1A), mRNA. |
| 3.6 | 1.0 | 1.3 | 1.0 | HSPA1A | heat shock 70kDa protein 1A (HSPA1A), mRNA. |
| 3.6 | 0.9 | 1.5 | 1.1 | SMTN | smoothelin (SMTN), transcript variant 3, mRNA. |
| 3.5 | 2.6 | 1.6 | 2.1 | DAF | decay accelerating factor for complement (CD55, Cromer blood group system) (DAF), mRNA. |
| 3.5 | 0.9 | 2.7 | 1.0 | ANTXR2 | anthrax toxin receptor 2 (ANTXR2), mRNA. |
| 3.5 | 1.0 | 1.3 | 0.9 | HSPA1A | heat shock 70kDa protein 1A (HSPA1A), mRNA. |
| 3.5 | 1.0 | 1.1 | 1.0 | HSPA1A | heat shock 70kDa protein 1A (HSPA1A), mRNA. |
| 3.4 | 1.5 | 1.8 | 1.6 | GNG11 | guanine nucleotide binding protein (G protein), gamma 11 (GNG11), mRNA. |
| 3.3 | 2.3 | 1.8 | 1.9 | PALM2-AKAP2 | PALM2-AKAP2 protein (PALM2-AKAP2), transcript variant 1, mRNA. |
| 3.3 | 2.1 | 1.8 | 1.8 | DDX10 | DEAD (Asp-Glu-Ala-Asp) box polypeptide 10 (DDX10), mRNA. |
| 3.3 | 1.9 | 1.4 | 1.3 | HSPA1A | heat shock 70kDa protein 1A (HSPA1A), mRNA. |
| 3.3 | 2.0 | 1.0 | 1.4 | C20orf51 | chromosome 20 open reading frame 51 (C20orf51), mRNA. |
| 3.3 | 1.4 | 1.5 | 1.7 | DRCTNNB1A | down-regulated by Ctnnb1, a (DRCTNNB1A), mRNA. |
| 3.2 | 2.0 | 1.5 | 1.5 | RRS1 | RRS1 ribosome biogenesis regulator homolog (S. cerevisiae) (RRS1), mRNA. |
| 3.2 | 1.0 | 1.2 | 1.0 | NUFIP1 | nuclear fragile X mental retardation protein interacting protein 1 (NUFIP1), mRNA. |
| 3.2 | 1.0 | 1.6 | 1.0 | CDCP1 | CUB domain-containing protein 1 (CDCP1), transcript variant 1, mRNA. |
| 3.2 | 2.6 | 2.2 | 1.9 | MGC17337 | similar to RIKEN cDNA 5730528L13 gene (MGC17337), mRNA. |
| 3.2 | 2.4 | 4.2 | 2.2 | PTX3 | pentaxin-related gene, rapidly induced by IL-1 beta (PTX3), mRNA. |
| 3.2 | 2.3 | 1.0 | 1.3 | CD22 | CD22 antigen |
| 3.1 | 2.1 | 1.3 | 1.5 | KRT1 | keratin 1 (epidermolytic hyperkeratosis) (KRT1), mRNA. |
| 3.1 | 2.1 | 1.4 | 1.4 | ISG20L1 | interferon stimulated exonuclease gene 20kDa-like 1 (ISG20L1), mRNA. |
| 3.1 | 2.4 | 1.9 | 1.8 | PPIF | peptidylprolyl isomerase F (cyclophilin F) (PPIF), nuclear gene encoding mitochondrial protein |
| 3.1 | 1.4 | 1.0 | 1.0 | INHBA | Inhibin, beta A (activin A, activin AB alpha polypeptide) |
| 3.0 | 2.1 | 1.8 | 1.8 | TSPAN5 | tetraspanin 5 (TSPAN5), mRNA. |
| 3.0 | 1.6 | 1.0 | 1.0 | KIAA1754 | KIAA1754 (KIAA1754), mRNA. |
| 3.0 | 2.6 | 1.6 | 2.0 | RTTN | rotatin (RTTN), mRNA. |
| 3.0 | 2.3 | 1.0 | 1.8 |  | Homo sapiens gag-pro-pol precursor protein gene, partial cds |
| 3.0 | 1.8 | 0.9 | 1.8 | CCDC10 | coiled-coil domain containing 10 (CCDC10), mRNA. |
| 3.0 | 2.0 | 1.2 | 1.4 | ADORA2B | adenosine A2b receptor (ADORA2B), mRNA. |
| 2.9 | 1.6 | 1.4 | 1.3 | CDYL | chromodomain protein, Y-like (CDYL), transcript variant 3, mRNA. |
| 2.9 | 2.2 | 0.8 | 1.9 |  | Homo sapiens SNAI1P pseudogene |
| 2.9 | 0.9 | 1.3 | 1.1 | GADD45B | growth arrest and DNA-damage-inducible, beta (GADD45B), mRNA. |
| 2.9 | 1.9 | 1.4 | 1.2 | DLC1 | deleted in liver cancer 1 (DLC1), transcript variant 2, mRNA. |
| 2.9 | 2.0 | 1.8 | 1.5 | GLIPR1 | GLI pathogenesis-related 1 (glioma) (GLIPR1), mRNA. |
| 2.9 | 2.6 | 1.2 | 2.0 | MME | membrane metallo-endopeptidase (neutral endopeptidase, enkephalinase, CALLA, CD10) (MME) |
| 2.9 | 1.3 | 1.6 | 1.1 | RAB27B | RAB27B, member RAS oncogene family (RAB27B), mRNA. |
| 2.9 | 3.0 | 1.8 | 1.8 | PHCA | phytoceramidase, alkaline (PHCA), mRNA. |
| 2.9 | 0.7 | 1.1 | 0.9 | UTP15 | UTP15, U3 small nucleolar ribonucleoprotein, homolog (yeast) (UTP15), mRNA. |
| 2.8 | 1.9 | 0.9 | 1.1 | SYNE1 | spectrin repeat containing, nuclear envelope 1 (SYNE1), transcript variant alpha, mRNA. |
| 2.8 | 1.0 | 1.4 | 1.0 | KPNA4 | karyopherin alpha 4 (importin alpha 3) (KPNA4), mRNA. |
| 2.8 | 1.7 | 1.4 | 1.5 | ANXA1 | annexin A1 (ANXA1), mRNA. |
| 2.7 | 2.0 | 1.8 | 1.6 | HPCAL1 | hippocalcin-like 1 (HPCAL1), transcript variant 1, mRNA. |
| 2.7 | 1.8 | 1.3 | 1.3 | TOMM34 | Translocase of outer mitochondrial membrane 34 |
| 2.7 | 2.7 | 1.5 | 1.6 | CDR2 | Cerebellar degeneration-related protein 2, 62kDa |
| 2.7 | 1.0 | 1.0 | 1.0 | SGNE1 | secretory granule, neuroendocrine protein 1 (7B2 protein) (SGNE1), mRNA. |
| 2.7 | 2.0 | 1.4 | 1.7 | CALM2 | calmodulin 2 (phosphorylase kinase, delta) (CALM2), mRNA. |
| 2.7 | 2.0 | 1.3 | 1.6 | LYAR | hypothetical protein FLJ20425 (LYAR), mRNA. |
| 2.7 | 2.4 | 1.0 | 1.9 | FLJ21687 | PDZ domain containing, X chromosome (FLJ21687), mRNA. |
| 2.6 | 2.4 | 1.0 | 2.2 |  | CDNA FLJ10171 fis, clone HEMBA1003807 |
| 2.6 | 2.1 | 1.3 | 1.2 | C1orf71 | chromosome 1 open reading frame 71 (C1orf71), mRNA. |
| 2.6 | 1.1 | 1.4 | 1.6 | CRY1 | cryptochrome 1 (photolyase-like) (CRY1), mRNA. |
| 2.6 | 1.6 | 1.0 | 1.8 | C1orf124 | chromosome 1 open reading frame 124 (C1orf124), transcript variant 2, mRNA. |
| 2.6 | 2.0 | 1.6 | 1.7 | SEC22L1 | SEC22 vesicle trafficking protein-like 1 (S. cerevisiae) (SEC22L1), mRNA. |
| 2.6 | 2.0 | 1.2 | 1.1 | CBLB | Cas-Br-M (murine) ecotropic retroviral transforming sequence b (CBLB), mRNA. |
| 2.6 | 2.4 | 1.5 | 2.0 | PPP1R15A | protein phosphatase 1, regulatory (inhibitor) subunit 15A (PPP1R15A), mRNA. |
| 2.5 | 1.6 | 0.7 | 1.0 | EMP1 | epithelial membrane protein 1 (EMP1), mRNA. |
| 2.5 | 1.0 | 1.4 | 0.6 | CAP1 | CAP, adenylate cyclase-associated protein 1 (yeast) (CAP1), mRNA. |
| 2.5 | 1.0 | 1.0 | 1.0 | ZCCHC6 | PREDICTED: Homo sapiens zinc finger, CCHC domain containing 6 (ZCCHC6), mRNA. |
| 2.5 | 2.0 | 2.1 | 1.9 |  | Human DNA sequence from clone RP3-527B10 on chromosome 6q25.1-25.3 |
| 2.5 | 1.0 | 1.5 | 1.0 | SLC39A14 | solute carrier family 39 (zinc transporter), member 14 (SLC39A14), mRNA. |
| 2.5 | 1.0 | 1.2 | 0.6 | SACS | spastic ataxia of Charlevoix-Saguenay (sacsin) (SACS), mRNA. |
| 2.5 | 1.0 | 1.4 | 1.0 | ATP13A3 | ATPase type 13A3 |
| 2.5 | 1.9 | 1.1 | 1.4 | ZXDB | zinc finger, X-linked, duplicated B (ZXDB), mRNA. |
| 2.5 | 1.0 | 1.3 | 0.8 | SAMD8 | sterile alpha motif domain containing 8 (SAMD8), mRNA. |
| 2.5 | 1.2 | 1.7 | 1.1 | HSPA8 | heat shock 70kDa protein 8 (HSPA8), transcript variant 1, mRNA. |
| 2.5 | 1.5 | 1.2 | 1.3 | STIP1 | stress-induced-phosphoprotein 1 (Hsp70/Hsp90-organizing protein) (STIP1), mRNA. |
| 2.5 | 1.4 | 1.4 | 1.5 | MKLN1 | Muskelin 1, intracellular mediator containing kelch motifs |
| 2.4 | 1.2 | 1.0 | 1.0 | KIAA0410 | KIAA0410 gene product |
| 2.4 | 1.7 | 1.0 | 1.6 | VIT | Vitrin |
| 2.4 | 1.0 | 1.7 | 0.8 | F2RL1 | coagulation factor II (thrombin) receptor-like 1 (F2RL1), mRNA. |
| 2.4 | 1.7 | 1.3 | 1.2 | LARP4 | La ribonucleoprotein domain family, member 4 (LARP4), transcript variant 1, mRNA. |
| 2.4 | 2.0 | 1.2 | 1.4 | PRICKLE1 | prickle-like 1 (Drosophila) (PRICKLE1), mRNA. |
| 2.4 | 3.1 | 1.8 | 2.0 | F3 | coagulation factor III (thromboplastin, tissue factor) (F3), mRNA. |
| 2.4 | 1.0 | 1.4 | 1.0 | HSPA4 | heat shock 70kDa protein 4 (HSPA4), transcript variant 1, mRNA. |
| 2.4 | 1.2 | 1.3 | 1.5 |  | Homo sapiens cDNA FLJ12327 fis, clone MAMMA1002140 |
| 2.4 | 2.0 | 1.0 | 2.1 | RP9 | retinitis pigmentosa 9 (autosomal dominant) (RP9), mRNA. |
| 2.4 | 1.4 | 1.4 | 1.4 | ZSWIM6 | Zinc finger, SWIM domain containing 6 |
| 2.4 | 1.9 | 1.3 | 1.6 | MGC2574 | hypothetical protein MGC2574 (MGC2574), mRNA. |
| 2.4 | 1.0 | 1.1 | 1.0 | MPHOSPH6 | M-phase phosphoprotein 6 (MPHOSPH6), mRNA. |
| 2.4 | 1.8 | 1.2 | 1.3 | TCP1 | t-complex 1 (TCP1), mRNA. |
| 2.3 | 1.9 | 1.4 | 1.8 | MCFD2 | multiple coagulation factor deficiency 2 (MCFD2), mRNA. |
| 2.3 |  |  |  | RGS19IP1 | GIPC PDZ domain containing family, member 1 |
| 2.3 | 1.0 | 1.4 | 1.2 | LOC56902 | putatative 28 kDa protein (LOC56902), mRNA. |
| 2.3 | 1.7 | 1.2 | 1.6 | PSMD14 | proteasome (prosome, macropain) 26S subunit, non-ATPase, 14 (PSMD14), mRNA. |
| 2.3 | 2.1 | 1.3 | 1.6 | UBE2N | ubiquitin-conjugating enzyme E2N (UBC13 homolog, yeast) (UBE2N), mRNA. |
| 2.3 | 1.5 | 1.5 | 1.1 | KCNN4 | potassium intermediate/small conductance calcium-activated channel, subfamily N, member 4 |
| 2.3 | 1.7 | 1.3 | 1.3 | STX3A | syntaxin 3A (STX3A), mRNA. |
| 2.3 | 2.0 | 1.1 | 1.4 | C14orf93 | chromosome 14 open reading frame 93 (C14orf93), mRNA. |
| 2.3 | 1.3 | 1.0 | 1.0 | SNAPC1 | small nuclear RNA activating complex, polypeptide 1, 43kDa (SNAPC1), mRNA. |
| 2.3 | 1.0 | 1.0 | 1.0 | DSU | dilute suppressor (DSU), mRNA. |
| 2.3 | 1.4 | 1.4 | 1.3 | HSPE1 | heat shock 10kDa protein 1 (chaperonin 10) (HSPE1), mRNA. |
| 2.3 | 1.6 | 1.2 | 1.4 | TIMM23 | translocase of inner mitochondrial membrane 23 homolog (yeast) (TIMM23), mRNA. |
| 2.3 | 2.1 | 1.0 | 1.3 | RP2 | retinitis pigmentosa 2 (X-linked recessive) (RP2), mRNA. |
| 2.3 | 1.3 | 1.6 | 1.4 | ANKHD1 | ankyrin repeat and KH domain containing 1 (ANKHD1), transcript variant 2, mRNA. |
| 2.3 | 1.8 | 1.3 | 1.5 | BET1 | BET1 homolog (S. cerevisiae) (BET1), mRNA. |
| 2.3 | 2.0 | 1.3 | 1.6 | GOLT1B | golgi transport 1 homolog B (S. cerevisiae) (GOLT1B), mRNA. |
| 2.3 | 1.9 | 1.8 | 1.7 | CD276 | CD276 antigen (CD276), transcript variant 2, mRNA. |
| 2.3 | 1.6 | 1.3 | 1.3 | DOCK9 | dedicator of cytokinesis 9 (DOCK9), mRNA. |
| 2.3 | 1.0 | 2.0 | 1.0 | FLJ23235 | hypothetical protein FLJ23235 (FLJ23235), mRNA. |
| 2.2 | 1.9 | 1.5 | 1.4 | NS5ATP13TP2 | NS5ATP13TP2 protein (NS5ATP13TP2), mRNA. |
| 2.2 | 2.2 | 1.4 | 1.5 | KPNA4 | karyopherin alpha 4 (importin alpha 3) (KPNA4), mRNA. |
| 2.2 | 2.3 | 1.5 | 1.7 | PSMD2 | proteasome (prosome, macropain) 26S subunit, non-ATPase, 2 (PSMD2), mRNA. |
| 2.2 | 0.8 | 1.3 | 0.7 | UBE2D1 | ubiquitin-conjugating enzyme E2D 1 (UBC4/5 homolog, yeast) (UBE2D1), mRNA. |
| 2.2 | 1.9 | 1.8 | 2.0 | QSCN6 | quiescin Q6 (QSCN6), transcript variant 1, mRNA. |
| 2.2 | 1.0 | 1.1 | 1.2 | NKIRAS1 | NFKB inhibitor interacting Ras-like 1 (NKIRAS1), mRNA. |
| 2.2 | 1.8 | 1.2 | 1.3 | DNAJB9 | DnaJ (Hsp40) homolog, subfamily B, member 9 (DNAJB9), mRNA. |
| 2.2 | 1.4 | 1.3 | 1.4 | CCT5 | chaperonin containing TCP1, subunit 5 (epsilon) (CCT5), mRNA. |
| 2.2 | 0.7 | 1.3 | 0.6 | FLJ13391 | hypothetical protein FLJ13391 (FLJ13391), mRNA. |
| 2.2 | 2.1 | 1.2 | 1.5 | GADD45A | growth arrest and DNA-damage-inducible, alpha (GADD45A), mRNA. |
| 2.2 | 2.1 | 1.8 | 2.4 | FLJ35036 | Hypothetical protein FLJ35036 |
| 2.2 | 1.6 | 1.2 | 1.5 | CGI-115 | CGI-115 protein (CGI-115), mRNA. |
| 2.2 | 1.8 | 1.4 | 1.6 | DDX21 | DEAD (Asp-Glu-Ala-Asp) box polypeptide 21 (DDX21), mRNA. |
| 2.2 | 1.7 | 1.2 | 1.3 | CCT6A | chaperonin containing TCP1, subunit 6A (zeta 1) (CCT6A), mRNA. |
| 2.1 | 1.8 | 1.2 | 1.2 | RAB8B | RAB8B, member RAS oncogene family |
| 2.1 | 1.7 | 1.2 | 1.4 | COX4NB | COX4 neighbor (COX4NB), mRNA. |
| 2.1 | 1.3 | 1.2 | 1.3 | ATPBD1B | ATP binding domain 1 family, member B (ATPBD1B), mRNA. |
| 2.1 |  |  |  | NARG1 | NMDA receptor regulated 1 (NARG1), mRNA. |
| 2.1 | 1.3 | 1.3 | 1.4 | PURA | purine-rich element binding protein A (PURA), mRNA. |
| 2.1 | 1.0 | 1.4 | 0.9 | PRO1855 | hypothetical protein PRO1855 (PRO1855), mRNA. |
| 2.1 | 1.6 | 1.3 | 1.4 | HSA9761 | putative dimethyladenosine transferase (HSA9761), mRNA. |
| 2.1 | 1.4 | 2.3 | 2.5 | IGSF4 | immunoglobulin superfamily, member 4 (IGSF4), mRNA. |
| 2.1 | 1.4 | 1.2 | 1.2 | ERBP | estrogen receptor binding protein (ERBP), mRNA. |
| 2.1 | 1.4 | 1.6 | 1.4 | LPIN2 | lipin 2 (LPIN2), mRNA. |
| 2.1 | 1.8 | 1.4 | 1.7 | KIAA0690 | KIAA0690 (KIAA0690), mRNA. |
| 2.0 | 1.3 | 1.3 | 1.4 | DRCTNNB1A | Down-regulated by Ctnnb1, a |
| 2.0 | 1.6 | 1.3 | 1.6 | SNRPA1 | small nuclear ribonucleoprotein polypeptide A' (SNRPA1), mRNA. |
|  |  |  |  |  |  |
|  |  |  |  |  | **Down-regulated genes** |
| **V12** | **S35** | **G37** | **C40** | **Gene** | **Description** |
| 26.9 | 9.8 | 6.2 | 8 | SYT8 | synaptotagmin VIII (SYT8), mRNA. |
| 25.6 | 9.5 | 18.7 | 8.2 | S100A14 | S100 calcium binding protein A14 (S100A14), mRNA. |
| 17 | 9.3 | 8.2 | 7.5 | TRIM29 | tripartite motif-containing 29 (TRIM29), transcript variant 2, mRNA. |
| 12.2 | 4.9 |  | 3.3 | MGC14436 | Hypothetical protein MGC14436 |
| 10.5 | 4.6 | 6 | 4.4 | TNNI2 | troponin I, skeletal, fast (TNNI2), mRNA. |
| 7.2 |  | 1.9 |  | LU | Lutheran blood group (Auberger b antigen included) (LU), mRNA. |
| 6.8 |  | 4.7 |  | SLPI | secretory leukocyte protease inhibitor (antileukoproteinase) (SLPI), mRNA. |
| 6.3 |  | 2.1 | 1.1 | AGR2 | anterior gradient 2 homolog (Xenopus laevis) (AGR2), mRNA. |
| 6.1 | 1.1 | 2 | 1.1 | UNC5B | unc-5 homolog B (C. elegans) (UNC5B), mRNA. |
| 5.8 | 2.3 | 2 | 2.3 | UNC5B | Unc-5 homolog B (C. elegans) |
| 5.7 | 2.1 |  | 1.2 | C9orf36 | chromosome 9 open reading frame 36 (C9orf36), mRNA. |
| 5.6 | 2.7 |  | 3.6 | C21orf62 | chromosome 21 open reading frame 62 (C21orf62), mRNA. |
| 5.1 | 4.8 | 1 | 2.3 | LOC203427 | similar to solute carrier family 25 , member 16 (LOC203427), mRNA. |
| 5.1 | 3.4 | 2.3 | 1.8 | TSC22D3 | TSC22 domain family, member 3 (TSC22D3), transcript variant 3, mRNA. |
| 4.9 | 2.3 | 2.6 | 2.3 | C20orf98 | chromosome 20 open reading frame 98 (C20orf98), mRNA. |
| 4.6 | 3.6 | 2.1 | 2 | CLU | clusterin (complement lysis inhibitor, SP-40,40, sulfated glycoprotein 2, apolipoprotein J) |
| 4.6 | 1.4 |  | 1.2 | NARG1L | NMDA receptor regulated 1-like |
| 4.5 |  | 2.3 |  | CLCA2 | chloride channel, calcium activated, family member 2 (CLCA2), mRNA. |
| 4.3 | 2.4 | 1.6 | 1.6 |  | Chromosome 7 open reading frame 19 |
| 4.1 |  | 2.1 | 1.1 | C9orf3 | chromosome 9 open reading frame 3 (C9orf3), mRNA. |
| 3.9 | 4.1 | 1.4 | 1.4 | C1S | complement component 1, s subcomponent (C1S), transcript variant 1, mRNA. |
| 3.8 | 2.2 | 1.5 | 1.3 | ZNF395 | zinc finger protein 395 (ZNF395), mRNA. |
| 3.8 |  | 1.8 |  | SPINT1 | serine protease inhibitor, Kunitz type 1 (SPINT1), transcript variant 2, mRNA. |
| 3.8 | 3 | 1.4 | 1.2 | C1R | complement component 1, r subcomponent (C1R), mRNA. |
| 3.8 | 2.6 |  | 1.6 | LOC401234 | PREDICTED: Homo sapiens hypothetical LOC401234 (LOC401234), mRNA. |
| 3.7 | 2.2 | 1.9 | 1.9 | EGFL3 | EGF-like-domain, multiple 3 |
| 3.7 | 1 | 2.2 | 1.1 | SERPINA1 | serine (or cysteine) proteinase inhibitor, clade A (alpha-1 antiproteinase), member 1, transcript var 3 |
| 3.5 | 2.6 | 1.6 | 1.7 | TXNIP | thioredoxin interacting protein (TXNIP), mRNA. |
| 3.4 | 1.9 | 2.3 | 1.3 | EPB72 | Erythrocyte membrane protein band 7.2 (stomatin) |
| 3.4 | 2.1 | 1.6 | 1.6 | SCNN1A | sodium channel, nonvoltage-gated 1 alpha (SCNN1A), mRNA. |
| 3.3 | 2.3 | 3.1 | 2.2 | GPNMB | glycoprotein (transmembrane) nmb (GPNMB), transcript variant 2, mRNA. |
| 3.3 | 2.1 | 1.6 | 1.4 | SLC2A4RG | SLC2A4 regulator (SLC2A4RG), mRNA. |
| 3.3 | 2.3 | 1.1 | 1.3 | C3 | complement component 3 (C3), mRNA. |
| 3.2 | 1.7 | 1.2 | 1.3 | KIAA0746 | KIAA0746 protein (KIAA0746), mRNA. |
| 3.2 | 1.2 |  | 1.1 |  | Homo sapiens, Similar to hypothetical protein FLJ20234, clone MGC:17335 IMAGE:4212810, mRNA |
| 3.2 | 2.2 |  | 1.9 | PCGEM1 | Prostate-specific non-coding gene |
| 3.2 | 2.3 | 2.1 | 2.2 | HPN | hepsin (transmembrane protease, serine 1) (HPN), transcript variant 2, mRNA. |
| 3 | 2.3 | 1.6 | 1.6 | HTRA1 | HtrA serine peptidase 1 (HTRA1), mRNA. |
| 3 | 2.1 | 1.5 | 1.3 | ZNF395 | zinc finger protein 395 (ZNF395), mRNA. |
| 3 |  | 1.7 |  |  | CDNA FLJ33090 fis, clone TRACH2000559 |
| 3 |  | 1.6 |  | SNCG | synuclein, gamma (breast cancer-specific protein 1) (SNCG), mRNA. |
| 2.9 | 1.3 | 1.9 | 1.1 | SYTL1 | synaptotagmin-like 1 (SYTL1), mRNA. |
| 2.9 | 1.5 | 1.7 | 1.5 | MGC15875 | Hypothetical protein MGC15875 |
| 2.8 | 1.7 | 1.6 | 1.3 | JUB | jub, ajuba homolog (Xenopus laevis) (JUB), transcript variant 2, mRNA. |
| 2.8 | 1.2 | 1.1 | 1.7 | LDLRAP1 | low density lipoprotein receptor adaptor protein 1 (LDLRAP1), mRNA. |
| 2.8 | 1.3 | 1.5 | 1.3 | CHES1 | Checkpoint suppressor 1 |
| 2.8 | 2.3 | 1.7 | 1.6 | KIAA0828 | KIAA0828 protein (KIAA0828), mRNA. |
| 2.8 | 1.7 | 1.8 | 1.2 | FAM46B | family with sequence similarity 46, member B (FAM46B), mRNA. |
| 2.8 |  | 1.4 |  | PEG10 | Paternally expressed 10 |
| 2.8 | 1.5 |  | 1.4 | CACNB2 | calcium channel, voltage-dependent, beta 2 subunit (CACNB2), transcript variant 8, mRNA. |
| 2.8 | 1.9 | 1.5 | 1.4 | CREG1 | cellular repressor of E1A-stimulated genes 1 (CREG1), mRNA. |
| 2.8 | 1.9 | 1.7 | 1.2 | ZBTB16 | zinc finger and BTB domain containing 16 (ZBTB16), mRNA. |
| 2.8 | 1.9 | 1.5 | 1.3 | TACSTD2 | tumor-associated calcium signal transducer 2 (TACSTD2), mRNA. |
| 2.7 | 1 |  | 1.1 | TTTY10 | testis-specific transcript, Y-linked 10 (TTTY10) on chromosome Y. |
| 2.7 | 1.2 | 1.9 | 1.3 | CPEB3 | cytoplasmic polyadenylation element binding protein 3 (CPEB3), mRNA. |
| 2.7 |  | 1.5 |  | SFRS1 | Splicing factor, arginine/serine-rich 1 (splicing factor 2, alternate splicing factor) |
| 2.7 | 1.6 | 1.4 | 1.4 | GPC1 | glypican 1 (GPC1), mRNA. |
| 2.7 | 1.7 | 2.5 | 1.8 | MTSS1 | metastasis suppressor 1 (MTSS1), mRNA. |
| 2.7 | 1.8 | 1.6 | 1.6 | BTBD6 | BTB (POZ) domain containing 6 (BTBD6), mRNA. |
| 2.7 | 2.1 | 1.9 | 1.8 | DDB2 | damage-specific DNA binding protein 2, 48kDa (DDB2), mRNA. |
| 2.7 | 1.3 | 2.1 | 1.4 | HR | Hairless homolog (mouse) |
| 2.7 | 1.5 |  | 1.2 | ALS2CR12 | Amyotrophic lateral sclerosis 2 (juvenile) chromosome region, candidate 12 |
| 2.6 | 1.4 | 1.4 | 1.6 | LTB4R | leukotriene B4 receptor (LTB4R), mRNA. |
| 2.6 | 1.7 | 1.5 | 1.3 | TPD52L1 | tumor protein D52-like 1 (TPD52L1), transcript variant 1, mRNA. |
| 2.6 | 1.8 | 1.4 | 1.6 | EEF1A2 | eukaryotic translation elongation factor 1 alpha 2 (EEF1A2), mRNA. |
| 2.6 | 2.3 | 1.9 | 1.6 | FKBP5 | FK506 binding protein 5 (FKBP5), mRNA. |
| 2.6 |  | 1.6 |  | PFAAP5 | Phosphonoformate immuno-associated protein 5 |
| 2.6 | 1.6 |  | 1.4 | DNA2L | PREDICTED: Homo sapiens DNA2 DNA replication helicase 2-like (yeast) (DNA2L), mRNA. |
| 2.6 | 1.4 |  | 1.2 |  | MRNA; cDNA DKFZp434A225 (from clone DKFZp434A225) |
| 2.6 | 6.9 | 5.9 | 3.5 | SNRPN | small nuclear ribonucleoprotein polypeptide N (SNRPN), transcript variant 1, mRNA. |
| 2.6 | 1.7 | 1.5 | 1.2 | SLC3A2 | Solute carrier family 3 (activators of dibasic and neutral amino acid transport), member 2 |
| 2.5 |  | 1.6 |  | APBB3 | amyloid beta (A4) precursor protein-binding, family B, member 3 (APBB3), transcript variant 1 |
| 2.5 | 2.8 | 1.8 | 2.2 | APOE | apolipoprotein E (APOE), mRNA. |
| 2.5 | 2.5 | 1.8 | 1.6 | FLJ40092 | FLJ40092 protein |
| 2.5 | 2.1 | 1.4 | 1.6 | TRIM16 | tripartite motif-containing 16 (TRIM16), mRNA. |
| 2.5 | 2.3 | 2.4 | 2.3 | MAL2 | mal, T-cell differentiation protein 2 (MAL2), mRNA. |
| 2.5 |  |  |  | HELZ | Helicase with zinc finger |
| 2.5 |  |  |  | LAMP3 | lysosomal-associated membrane protein 3 (LAMP3), mRNA. |
| 2.5 | 1.7 | 1.5 | 1.2 | FLJ12684 | Hypothetical protein FLJ12684 |
| 2.5 | 2.5 |  | 1.9 | SLC7A7 | solute carrier family 7 (cationic amino acid transporter, y+ system), member 7 (SLC7A7), mRNA. |
| 2.4 | 1 | 1.2 | 1.2 | PRSS7 | protease, serine, 7 (enterokinase) (PRSS7), mRNA. |
| 2.4 | 1.5 | 1.3 | 1.3 | CBX4 | chromobox homolog 4 (Pc class homolog, Drosophila) (CBX4), mRNA. |
| 2.4 | 1.4 | 1.7 | 1.3 | FLJ14054 | hypothetical protein FLJ14054 (FLJ14054), mRNA. |
| 2.4 | 2.5 | 1.6 | 2 | PRO1073 | PRO1073 protein |
| 2.4 | 1.7 | 2.2 | 2 | TSPAN-1 | tetraspan 1 (TSPAN-1), mRNA. |
| 2.4 | 1.4 | 1.1 | 1.4 | IFI30 | interferon, gamma-inducible protein 30 (IFI30), mRNA. |
| 2.4 |  | 1.2 |  | MN1 | meningioma (disrupted in balanced translocation) 1 (MN1), mRNA. |
| 2.4 | 1.6 | 1.5 | 1.5 | PVRL1 | Poliovirus receptor-related 1 (herpesvirus entry mediator C; nectin) |
| 2.4 | 1.8 | 1.9 | 1.5 | TRERF1 | transcriptional regulating factor 1 (TRERF1), transcript variant 2, mRNA. |
| 2.4 | 2.2 | 2.3 | 1.8 | C14orf78 | PREDICTED: Homo sapiens chromosome 14 open reading frame 78 (C14orf78), mRNA. |
| 2.4 |  |  |  | NRBP2 | nuclear receptor binding protein 2 (NRBP2), mRNA. |
| 2.4 |  | 1.4 | 1 | TMEM14A | transmembrane protein 14A (TMEM14A), mRNA. |
| 2.4 | 1.2 |  | 1.4 | TBC1D5 | TBC1 domain family, member 5 |
| 2.4 | 1.2 | 1.7 | 1.2 | LRP1 | low density lipoprotein-related protein 1 (alpha-2-macroglobulin receptor) (LRP1), mRNA. |
| 2.3 | 1.8 |  | 1.1 | CECR2 | Cat eye syndrome chromosome region, candidate 2 |
| 2.3 | 1.3 |  | 1.6 | HSPA14 | Heat shock 70kDa protein 14 |
| 2.3 |  | 1.4 |  | AP1G2 | adaptor-related protein complex 1, gamma 2 subunit (AP1G2), transcript variant 1, mRNA. |
| 2.3 | 1.8 | 1.9 | 1.5 | LOC92771 | Similar to MYOSIN HEAVY CHAIN, CARDIAC MUSCLE ALPHA ISOFORM (MYHC-ALPHA) |
| 2.3 | 1.5 |  | 1.3 |  | CDNA FLJ26539 fis, clone KDN09310 |
| 2.3 | 2.1 |  | 2.1 | FLJ22671 | hypothetical protein FLJ22671 (FLJ22671), mRNA. |
| 2.3 | 1.6 | 1.3 | 1.1 | SNN | stannin (SNN), mRNA. |
| 2.3 | 1.7 |  | 1.3 | POLDIP3 | Polymerase (DNA-directed), delta interacting protein 3 |
| 2.3 |  |  |  | MCLC | Chloride channel CLIC-like 1 |
| 2.3 | 1.6 | 1.5 | 1.3 | LOC375295 | PREDICTED: Homo sapiens hypothetical gene supported by BC013438 (LOC375295), mRNA. |
| 2.3 |  | 1.5 |  |  | CDNA: FLJ21274 fis, clone COL01781 |
| 2.2 | 1.4 | 1.5 | 1.2 | PRSS12 | protease, serine, 12 (neurotrypsin, motopsin) (PRSS12), mRNA. |
| 2.2 | 1.9 | 1.4 | 1.4 | RNPC3 | RNA-binding region (RNP1, RRM) containing 3 (RNPC3), mRNA. |
| 2.2 | 1.8 | 1.5 | 1.3 | FLJ12436 | hypothetical protein FLJ12436 (FLJ12436), mRNA. |
| 2.2 |  | 1.1 |  | MGC16121 | Hypothetical protein MGC16121 |
| 2.2 | 1.1 | 1.5 | 1 | LOC92154 | Hypothetical protein BC002770 |
| 2.2 | 1.5 | 1.2 | 1.2 | PER1 | period homolog 1 (Drosophila) (PER1), mRNA. |
| 2.2 | 1.3 |  | 1.1 | ZNF485 | zinc finger protein 485 (ZNF485), mRNA. |
| 2.2 | 1.4 | 1.8 | 1.5 | MJD | Machado-Joseph disease (spinocerebellar ataxia 3, olivopontocerebellar ataxia 3, autosomal domin |
| 2.2 | 1.8 | 1.9 | 1.4 | FBXO32 | F-box protein 32 (FBXO32), transcript variant 2, mRNA. |
| 2.1 | 1.6 | 1.3 | 1.3 | IGSF3 | Immunoglobulin superfamily, member 3 |
| 2.1 | 1.9 | 1.9 | 1.7 | SSBP2 | single-stranded DNA binding protein 2 (SSBP2), mRNA. |
| 2.1 |  | 1.2 |  | NDRG2 | NDRG family member 2 (NDRG2), transcript variant 5, mRNA. |
| 2.1 | 1.3 |  | 1.2 | ZBTB7B | zinc finger and BTB domain containing 7B (ZBTB7B), mRNA. |
| 2.1 | 1.5 | 1.1 | 1.2 | KIAA0922 | KIAA0922 protein (KIAA0922), mRNA. |
| 2.1 | 1.4 | 1.3 | 1.3 | TNKS1BP1 | tankyrase 1 binding protein 1, 182kDa (TNKS1BP1), mRNA. |
| 2.1 |  | 1.2 |  | PTGFRN | prostaglandin F2 receptor negative regulator (PTGFRN), mRNA. |
| 2.1 |  | 1.2 | 1.7 | C5orf13 | chromosome 5 open reading frame 13 (C5orf13), mRNA. |
|  |  |  |  |  |  |
|  |  |  |  |  |  |
|  |  |  |  |  | **Transcripton factors/chromatin remodeling** |
|  |  |  |  |  |  |
|  |  |  |  |  | **Up-regulated genes** |
| **V12** | **S35** | **G37** | **C40** | **Gene** | **Description** |
| 9.1 | 8.0 | 6.1 | 6.2 | ETV5 | ets variant gene 5 (ets-related molecule) (ETV5), mRNA. |
| 5.5 | 2.1 | 1.0 | 1.6 | FOXO1A | Forkhead box O1A (rhabdomyosarcoma) |
| 4.7 | 2.9 | 1.9 | 2.1 | ETS1 | v-ets erythroblastosis virus E26 oncogene homolog 1 (avian) (ETS1), mRNA. |
| 4.1 | 2.9 | 2.1 | 2.4 | HMGA2 | high mobility group AT-hook 2 (HMGA2), mRNA. |
| 3.5 | 1.7 | 1.5 | 1.2 | SOX9 | SRY (sex determining region Y)-box 9 (campomelic dysplasia, autosomal sex-reversal) (SOX9) |
| 3.5 | 2.0 | 1.2 | 1.2 | KLF6 | Kruppel-like factor 6 (KLF6), transcript variant 2, mRNA. |
| 3.3 |  |  |  | KLF6 | Kruppel-like factor 6 (KLF6), transcript variant 2, mRNA. |
| 3.3 | 2.3 | 2.2 | 2.1 | IER3 | immediate early response 3 (IER3), transcript variant short, mRNA. |
| 2.9 | 1.7 | 1.1 | 1.2 | ZFP36L1 | zinc finger protein 36, C3H type-like 1 (ZFP36L1), mRNA. |
| 2.8 | 2.4 | 2.3 | 2.0 | MAFF | v-maf musculoaponeurotic fibrosarcoma oncogene homolog F (avian) (MAFF), transcript variant 2 |
| 2.5 | 0.3 | 1.0 | 0.5 | RRN3 | RRN3 RNA polymerase I transcription factor homolog (yeast) (RRN3), mRNA. |
| 2.5 | 2.9 | 1.0 | 1.4 | BHLHB2 | basic helix-loop-helix domain containing, class B, 2 (BHLHB2), mRNA. |
| 2.3 | 1.2 | 1.2 | 1.1 | DLX2 | distal-less homeo box 2 (DLX2), mRNA. |
| 2.3 | 1.6 | 1.2 | 1.3 | HBXAP | hepatitis B virus x associated protein (HBXAP), mRNA. |
| 2.3 | 1.6 | 1.1 | 1.3 | MYC | v-myc myelocytomatosis viral oncogene homolog (avian) (MYC), mRNA. |
| 2.2 | 1.5 | 1.1 | 1.1 | NR3C1 | nuclear receptor subfamily 3, group C, member 1 (glucocorticoid receptor) (NR3C1), mRNA. |
| 2.2 | 2.2 | 1.3 | 2.5 | AHR | aryl hydrocarbon receptor (AHR), mRNA. |
| 2.1 | 1.7 | 1.2 | 1.6 | CITED2 | Cbp/p300-interacting transactivator, with Glu/Asp-rich carboxy-terminal domain, 2 (CITED2), mRNA. |
| 2.1 | 1.5 | 1.7 | 1.5 | SMARCA4 | SWI/SNF related, matrix associated, actin dependent regulator of chromatin, subfamily a, member 4 |
| 2.1 | 1.0 | 1.1 | 1.0 | MXD1 | MAX dimerization protein 1 (MXD1), mRNA. |
| 2.1 | 2.5 | 1.4 | 2.1 | WWTR1 | WW domain containing transcription regulator 1 |
| 2.0 | 2.1 | 1.3 | 1.3 | ZBED4 | Zinc finger, BED domain containing 4 |
| 2.0 | 1.7 | 1.4 | 1.4 | PHC2 | polyhomeotic-like 2 (Drosophila) (PHC2), transcript variant 2, mRNA. |
|  |  |  |  |  |  |
|  |  |  |  |  |  |
|  |  |  |  |  | **Down-regulated genes** |
| **V12** | **S35** | **G37** | **C40** | **Gene** | **Description** |
| 6.6 | 1.6 | 2.1 | 1.2 | PIR | pirin (iron-binding nuclear protein) (PIR), mRNA. |
| 4 | 2.6 |  | 1.9 | ZIC5 | Zic family member 5 (odd-paired homolog, Drosophila) (ZIC5), mRNA. |
| 3.5 | 1.1 | 2.8 | 1.2 | H1F0 | H1 histone family, member 0 (H1F0), mRNA. |
| 3.2 | 2.3 | 1.8 | 1.9 | HIST1H2AC | Histone 1, H2ac |
| 3.1 | 2.3 | 2.2 | 2.2 | HIST2H2AA | histone 2, H2aa (HIST2H2AA), mRNA. |
| 3.1 | 1.4 |  | 1.1 | MLL2 | myeloid/lymphoid or mixed-lineage leukemia 2 (MLL2), mRNA. |
| 2.8 | 1.7 | 1.4 | 1.6 | POU2AF1 | POU domain, class 2, associating factor 1 (POU2AF1), mRNA. |
| 2.7 | 1.9 | 1.5 | 1.5 | MAFB | v-maf musculoaponeurotic fibrosarcoma oncogene homolog B (avian) (MAFB), mRNA. |
| 2.4 |  |  |  | ZIK1 | zinc finger protein interacting with K protein 1 (ZIK1), mRNA. |
| 2.3 | 1.7 | 1.2 | 1.1 | CEBPD | CCAAT/enhancer binding protein (C/EBP), delta (CEBPD), mRNA. |
| 2.3 | 1.7 |  | 1.2 | TFEB | transcription factor EB (TFEB), mRNA. |
| 2.2 |  | 1.4 |  | MXD4 | MAX dimerization protein 4 (MXD4), mRNA. |
| 2.2 | 1.5 |  | 1.3 | CNOT4 | CCR4-NOT transcription complex, subunit 4 |
