# Supplementary material for: TDAG51 is an ERK signaling target that opposes ERK-mediated HME16C mammary epithelial cell transformation
Source: BMC Cancer. 2008 Jul 2;8:189. doi: 10.1186/1471-2407-8-189 (PMC2474852; doi:10.1186/1471-2407-8-189)
Supplement: Additional file 2 — Real-time RT-PCR confirmation of selected microarray data. Results are for transcripts identified as up- or down-regulated in microarray analysis of cDNA from RasV12-, RasV12G37-, RasV12S35-, RasV12C40-, and Rlf-CAAX-infected HME16C. Numbers represent fold expression changes for transcript levels relative to vector-infected pLRT control cells. Parenthetical numbers indicate mean fold expression changes from cDNA microarray analysis (see Additional file above). Sequences of primers used for real-time RT-PCR analysis are as follows: amphiregulin Forward 5'-CGCCGGTGGTGCTGTCGCTCTT-3' Reverse 5'-TCACTCACAGGGGAAATCTCACTC-3', anterior gradient 2 Forward 5'-GGGGTGACCAACTCATCTGGACTCAG-3' Reverse 5'-GACATACTGGCCATCAGGAGAAAGGTGT-3', CD24 Forward 5'-GCTCCTACCCACGCAGATTTATT-3' Reverse 5'-CACGAAGAGACTGGCTGTTGACT-3', c-met Forward 5'-AAATGGCCACGGGACAACACAA-3' Reverse 5'-TGGGCTGGGGTATAACATTCAAGA-3', CTGF Forward 5'-CTGCCCGGGAAATGCTGCGAGGAGT-3' Reverse 5'-CTGCAGGAGGCGTTGTCATTGGTAA-3', CYR61 Forward 5'-CGGCCCAAGTACTGCGGTTCCT-3' Reverse 5'-ATTGGCATGCGGGCAGTTGTAGTT-3', Decorin Forward 5'-ACTTCTGCCCACCTGGACACAACA-3' Reverse 5'-ATGGCAGAGCGCACGTAGACACA-3', DICER-1 Forward 5'-CAGGAAATACCCGTGCAACCAACTA-3' Reverse 5'-GCATTACGGCCATCACAGGACTTC-3', E-cadherin Forward 5'-GGTATCTTCCCCGCCCTGCCAATCC-3' Reverse 5'-AACCGCTTCCTTCATAGTCAAACACGAG-3', EphA2 Forward 5'-CCCCTTCCGCCCCACACTACCTCACAGC-3' Reverse 5'-ACACGGCCCGCATTCCCCAGACTCG-3', Epiregulin Forward 5'-TTGTATTTTTAGTAGAGGCGGGGTTTCA-3' Reverse 5'-TCGGGCACAGATGTTCAAGTCAC-3', ETS-1 Forward 5'-ACTCGGGGGCCAGGACTCTTTTGAA-3' Reverse 5'-CACGGTCCCGCACATAGTCCTTGAA-3', ETS variant gene 5 Forward 5'-TCGGGGACGTCTACGGTTTCTACT-3' Reverse 5'-AAGACTGTAAACGGCTACCATTGA-3', FAT-2 Forward 5'-GCTGGACATCAAACGGGCTAACAT-3' Reverse 5'-ACCGCATCTGAACCCCCACTGAAT-3', HMGA-1 Forward 5'-GCTCACCCTGCCCGCTCCCAACC-3' Reverse 5'-GCCCCAGCCCCTCTTCCCCACAAA-3', HMGA-2 Forward 5'-CTGATAAGCAAGAGTGGGCGGGTGAGAA-3' Reverse 5'-ACAGGGAGTGGGTTGGGGTGGTATTTGA-3', Secreted frizzled-related protein [file 1471-2407-8-189-S2.doc]

|  | HME16 Cell Line Fold Upregulation | | | | |
| --- | --- | --- | --- | --- | --- |
| Gene Name | **V12** | **G37** | **S35** | **C40** | **Rlf-CAAX** |
| Amphiregulin | **1.4** (2.4) | **2.3** (1.8) | **2.9** (ND) | **2.2** (ND) | **1.3** (1.2) |
| Anterior gradient 2 | **-2.6** (-6.3) | **-2.2** (-2.1) | **-1.7** (ND) | **-3.0** (1.0) | **-4.9** (-2.3) |
| CD24 | **-7.6** (ND) | **-4.1** (-5.5) | **-4.9** (-3.9) | **-3.7** (-3.8) | **-5.2** (1.0) |
| c-met | **7.9** (3.4) | **1.6** (1.6) | **6.6** (2.4) | **3.0** (1.4) | **1.7** (1.2) |
| CTGF | **8.4** (5.9) | **3.7** (3.6) | **7.5** (4.2) | **4.4** (3.1) | **2.9** (3.0) |
| CYR61 | **3.1** (2.7) | **2.1** (2.6) | **3.1** (2.8) | **3.1** (2.2) | **1.7** (2.1) |
| Decorin | **-2.9** (-9.9) | **-3.8** (ND) | **-8.7** (-6.5) | **-6.3** (-3.8) | **-2.9** (ND) |
| DICER-1 | **1.3** (1.4) | **-1.7** (1.1) | **3.4** (2.1) | **1.8** (1.7) | **1.3** (1.0) |
| E-cadherin | -**12.5** (-5.7) | **-4.3** (1.0) | **-7.8** (-1.3) | **-5.5** (-1.2) | **-1.4** (-1.8) |
| EphA2 | **5.8** (4.2) | **2.1** (2.2) | **4.5** (4.0) | **2.5** (2.0) | **1.0** (1.3) |
| Epiregulin | **2.6** (3.6) | **1.8** (2.4) | **1.9** (ND) | **1.2** (1.3) | **1.3** (ND) |
| ETS-1 | **8.2** (4.7) | **3.0** (1.9) | **5.6** (3.2) | **4.1** (2.2) | **1.9** (1.3) |
| ETS varient gene 5 | **10.7** (9.1) | **8.9** (6.1) | **12.9** (8.0) | **9.4** (6.2) | **-1.3** (ND) |
| FAT-2 | **-5.2** (-11.1) | **-2.8** (-2.4) | **-4.9** (-4.3) | **-2.5** (-2.2) | **1.2** (-1.7) |
| HMGA-1 | **1.6** (2.2) | **1.6** (2.7) | **1.2** (1.1) | **1.0** (1.2) | **1.1** (1.1) |
| HMGA-2 | **4.0** (4.2) | **2.3** (2.1) | **4.7** (3.0) | **3.7** (2.4) | **1.9** (1.3) |
| Secreted frizzled-  related protein 1 | **-2.7** (-3.0) | **-2.3** (-1.9) | **-2.1** (-2.0) | **-1.7** (-1.8) | **1.0** (-1.4) |
| Tenascin C | **2.9** (3.1) | **2.2** (3.1) | **4.3** (ND) | **2.1** (ND) | **1.0** (1.4) |
| TGF-alpha | **3.5** (1.9) | **-1.1** (1.3) | **2.9** (1.7) | **1.9** (1.3) | **-1.5** (1.2) |
| TGF-beta2 | **2.1** (1.5) | **1.0** (2.0) | **4.1** (1.1) | **3.3** (1.3) | **-2.0** (1.4) |
| TGF-beta receptor 2 | **3.8** (3.4) | **2.1** (-1.4) | **4.3** (1.3) | **2.7** (1.0) | **2.3** (ND) |
| TRIM29 | **-38.0** (-17.0) | **-9.3** (-8.2) | **-36.0** (-9.3) | **-9.3** (-7.5) | **-1.2** (-1.6) |
